# The LIM Domain Protein nTRIP6 Recruits the Mediator Complex to AP-1-Regulated Promoters

**DOI:** 10.1371/journal.pone.0097549

**Published:** 2014-05-12

**Authors:** Markus E. Diefenbacher, Daniela Reich, Oliver Dahley, Denise Kemler, Margarethe Litfin, Peter Herrlich, Olivier Kassel

**Affiliations:** 1 Karlsruhe Institute of Technology (KIT), Institute of Toxicology and Genetics, Karlsruhe, Germany; 2 Leibniz Institute for Age Research (Fritz Lipmann Institute), Jena, Germany; University of Birmingham, United Kingdom

## Abstract

Several LIM domain proteins regulate transcription. They are thought to act through their LIM protein-protein interaction domains as adaptors for the recruitment of transcriptional co-regulators. An intriguing example is nTRIP6, the nuclear isoform of the focal adhesion protein TRIP6. nTRIP6 interacts with AP-1 and enhances its transcriptional activity. nTRIP6 is also essential for the transrepression of AP-1 by the glucocorticoid receptor (GR), by mediating GR tethering to promoter-bound AP-1. Here we report on the molecular mechanism by which nTRIP6 exerts these effects. Both the LIM domains and the pre-LIM region of nTRIP6 are necessary for its co-activator function for AP-1. Discrete domains within the pre-LIM region mediate the dimerization of nTRIP6 at the promoter, which enables the recruitment of the Mediator complex subunits THRAP3 and Med1. This recruitment is blocked by GR, through a competition between GR and THRAP3 for the interaction with the LIM domains of nTRIP6. Thus, nTRIP6 both positively and negatively regulates transcription by orchestrating the recruitment of the Mediator complex to AP-1-regulated promoters.

## Introduction

After binding to their cognate DNA response elements in the regulatory regions of their target genes, sequence-specific transcription factors activate transcription through the recruitment of transcriptional co-activators. Co-activators are defined as factors which do not directly bind DNA but are recruited to promoters through a direct or indirect interaction with DNA-binding transcription factors, and which participate in the activation of transcription. Co-activators are generally classified according to the mechanism by which they promote transcriptional activation (reviewed in [1], [2]). This can be for example the modification of histone tails, the remodelling of nucleosomes, or the recruitment and stimulation of the basal transcription machinery. These different functions are dynamically coordinated through the sequential recruitment and release of specific co-activators or multi-protein co-activator complexes at the promoter [reviewed in 3,4]. A prominent role in this orchestration is that played by adaptor proteins. These proteins formally match the definition of co-activators, in that they are recruited to promoters via an interaction with transcription factors and are involved in transcriptional activation. However their role is to recruit other factors which function as co-activators. One of the early examples of such an adaptor protein is CBP/p300. Although this large protein acts as a co-activator through its intrinsic histone acetyl-transferase activity [5], [6], it also serves as an adaptor protein, by interacting with other essential co-activators such as SRC-1 or p/CAF (reviewed in [7], [8]), thus enabling the recruitment of larger co-activator complexes. Other co-activators act only as adaptor proteins. This is the case for a group of LIM domain-containing co-activators (reviewed in [9], [10]). The LIM domain, first identified in the Lin-11, Isl-1 and Mec-3 homeodomain transcription factors, is defined as a cysteine-rich motif organized as a double zinc finger structure, which mediates protein-protein interactions. Some LIM domain proteins consist of nearly exclusively LIM domains (LIM-only proteins), and yet act as co-activators. For example, the Four-and-a-Half LIM domain protein 2 (FHL2) acts as a co-activator for the androgen receptor [11] and for CREB [12], whereas the LIM-only protein 4 (LMO4), which consists of only 2 LIM domains, co-activates Peroxisome Proliferator-Activated Receptor-γ [13] and Smad4 [14]. Given that the only functional domains in these LIM proteins are protein-protein interaction modules, the only way they can act on transcription is as adaptors recruiting other co-activators.

A particularly intriguing class of LIM domain proteins are members of the Zyxin and Paxillin families of so-called focal adhesion LIM domain proteins. The proteins of this group are cytosolic, enriched at sites of focal adhesion, and are known to regulate adhesion and migration (reviewed in [10]). Surprisingly, LIM domain proteins of this class also exert co-activator or co-repressor functions for various transcription factors, and have thus been proposed to shuttle from the cytoplasm to the nucleus to regulate transcription (reviewed in [15], [16]). Amongst these proteins a particular case is that of TRIP6 (reviewed in [17], [18]). We have reported that this protein is not shuttling, but that its nuclear functions are mediated by a shorter, exclusively nuclear isoform, which we termed nTRIP6 [19]. nTRIP6 acts as a co-activator for the transcription factors AP-1, NF-κB and the glucocorticoid receptor (GR). nTRIP6 interacts with these transcription factors, is recruited to the transcription factor-bound target promoters via this interaction, and is required for the activation of transcription [19]–[21]. Thus, nTRIP6 fulfils the definition of a co-activator. Like the other members of the Zyxin family of LIM domain proteins, nTRIP6 harbours three C-terminal LIM domains [22] with interaction specificities for the transcription factors mentioned above [19]. The N-terminal pre-LIM region does not contain any known functional domains which could account for the co-activator function. Therefore, we favour the hypothesis that nTRIP6 acts as an adaptor protein, which promotes the recruitment of other co-activators or co-activator complexes to transcription factor-bound promoters.

Another essential function of nTRIP6 is to mediate the repressive crosstalk between AP-1 and GR, a phenomenon referred to as transrepression [23]–[28]. In this mode of action, GR does not act as a transcription factor but rather as a co-repressor for AP-1, in that it is tethered to the AP-1-bound promoter through protein-protein interactions, leading to transcriptional repression [19], [29]–[32]. We have previously reported that through the selectivity of its LIM domains, nTRIP6 mediates the tethering of GR to the promoter of AP-1 target genes [19], [20]. However, the mechanism by which this nTRIP6-dependent tethering leads to repression has remained unclear.

We report here that unexpectedly, both the pre-LIM region and the LIM domains of nTRIP6 are required for its co-activator function. nTRIP6 homodimerizes through discrete domains within its pre-LIM region, and this homodimerization is required for the co-activator function of nTRIP6 for AP-1. Mechanistically, our data demonstrate that nTRIP6 homodimerization enables the recruitment of the Mediator complex subunit THRAP3 to the promoters, through the interaction of THRAP3 with the LIM domains. Furthermore, we show that GR prevents the interaction between nTRIP6 and THRAP3, inhibits THRAP3 recruitment to AP-1 target promoters, and thereby represses AP-1 activity. These data document a critical adaptor function for nTRIP6 in the regulation of transcription.

## Materials and Methods

### Plasmid constructs

The luciferase reporter constructs were as previously described: MMP1-Luc [33], GAL-Luc [34] and Ubi-Renilla [19]. pcDNA3.1HA-nTRIP6 has been described [19]. pcDNA3.1HA-preLIM and pcDNA3.1HA-LIM were constructed by PCR amplification of the sequence encoding the nTRIP6 pre-LIM region, lacking the LIM domains, and of the LIM domains of TRIP6, respectively, and subcloning into pcDNA3.1HA [19]. The GAL4 DNA binding domains fusions of nTRIP6, preLIM and LIM were generated by PCR and subcloning into pcDNAGal_DBD_ [19]. The constructs for bimolecular fluorescence complementation assays (BiFC; [35], [36]) were cloned as follows: the C- and N-terminal halves of the Venus fluorescent protein (provided by Chang-Deng Hu, Purdue University, West Lafayette IN) were PCR-amplified and cloned between the NotI and XbaI sites of pcDNA3.1HA to obtain pcDNA3.1HA-VC and pcDNA3.1HA-VN, respectively. pcDNA3.1HA-nTRIP6-VC, pcDNA3.1HA-nTRIP6-VN, pcDNA3.1HA-preLIM-VC and pcDNA3.1HA-LIM-VC were obtained by cloning the corresponding PCR fragments into pcDNA3.1HA-VC or pcDNA3.1HA-VN. The BiFC deletion constructs lacking either the dimerization domain 1 (DD1) or the dimerization domain 2 (DD2) were generated using the In-Fusion HD cloning kit (Clontech, Saint-Germain-en-Laye, France) according to the manufacturer's instructions, and cloned into pcDNA3.1HA-VC and pcDNA3.1HA-VN. The fusions to the C-terminal half of YFP (YC) of nTRIP6 and its LIM mutants, in which the coordinating cysteines of the two zinc fingers of either the first or the third LIM domains were mutated to alanines (LIM1 and LIM3m), have been described [21]. Similarly, in pcDNA3.1-nTRIP6-LIM2m-YC, the first two coordinating cysteines in both zinc fingers of the second LIM domain were mutated to alanine using the QuickChange Site-Directed mutagenesis kit (Stratagene, Heidelberg, Germany). pcDNA-mCherry-NES and pGEX-4T-3-nTRIP6 for GST-nTRIP6 expression have been described [21]. The pCG expression vectors for the single-chain AP-1 constructs c-Jun∼ATF2 and c-Jun∼c-Fos [37] were from Latifa Bakiri (Spanish National Cancer Center, Madrid, Spain). The mCherry-tagged, nuclear targeted blocking peptides DD1 and DD2, as well as their scrambled control versions DD1c and DD2c, were obtained by cloning mCherry (provided by Roger Y. Tsien, University of California, San Diego, La Jolla CA) between the NheI and KpnI sites, an oligonucleotide encoding the nuclear localization signal of the SV40 virus between the KpnI and BamHI sites, and oligonucleotides encoding the peptides between the BamHI and XbaI sites of pcDNA3.1. The THRAP3 (TRAP150) expression vector pcDNA-TRAP150-FLAG [38] was a gift from Woan-yuh Tarn (National Taiwan University, Taipei, Taiwan). For BiFC experiments, THRAP3 was fused with the N-terminal half of YFP (YN), using the In-Fusion cloning kit, and cloned into pcDNA3.1. pΔBN-AR1 [39] for the generation of the MMP1-Luc array cell line was obtained from Noriaki Shimizu (Hiroshima University, Hiroshima, Japan). pcDNA3.1-GR has been described [19].

### Cell culture and transfections

HeLa, HEK-293, Cos7 and NIH-3T3 fibroblasts (ATCC, LGC Standards GmbH, Wesel; Germany) were cultured in Dulbecco's modified Eagle's medium (DMEM) supplemented with 10% fetal calf serum. For reporter gene assays and BiFC experiments, cells were transfected using PromoFectin (PromoKine, Heidelberg, Germany). For mRNA analysis cells were transfected using Screenfect (Incella, Graben-Neudorf, Germany).

Synthetic siRNA duplexes were purchased from Eurofins MWG Operon (Ebersberg Germany). HEK-293 cells were transfected with a mixture of two siRNAs targeting THRAP3 mRNA (sequence as in [40]), or a control siRNA targeting dsRed [41] using Lipofectamine 2000 (Invitrogen GmbH, Karlsruhe, Germany).

Stable NIH-3T3 clones bearing an array of amplified MMP1-Luc gene unit were obtained by blasticidin selection after cotransfection of equimolar amounts of MMP1-Luc and pΔBN-AR1, which promotes an amplification of cotransfected plasmids [39]. Copy number was estimated as previously described [20], [21], [42] by real-time PCR analysis, using primers amplifying both the Chinese hamster DHFR genomic region contained in pΔBN-AR1 and the endogenous mouse counterpart. The presence of the array was confirmed by fluorescence in situ hybridization (FISH), as described ([20], [21]; see Material and Methods S1). The stable array cells were further transfected using JetPEI (PEQLAB Biotechnologie, Erlangen Germany).

All experiments were performed in serum-starved (24 h) cells. Unless otherwise stated, cells were treated 24 h post-transfection with solvent alone or 50 ng/ml TPA (Sigma-Aldrich, Munich, Germany), in the presence or absence of 1 µM dexamethasone (Sigma-Aldrich). In siRNA experiments, cells were treated 48 h post-transfection. For reporter gene assays, cells were harvested 16 h post-treatment. Except for the experiments with Cos7 cells overexpressing THRAP3, firefly luciferase activities were normalized to Renilla luciferase activities (Ubi-Renilla). Cells were harvested 4 h after TPA treatment for mRNA analysis, or 3 h after TPA treatment for chromatin immunoprecipitation and array cells imaging.

### Western blotting

Western blot analyses were performed using the following antibodies: anti-THRAP3 (Novus Biologicals, Littleton, CO); anti-c-Fos (Upstate, Schwalbach, Germany); anti-HA (clone 3F10, Roche Applied Science, Mannheim, Germany); anti-GR (clone 4H2; Novocastra); anti-actin (Santa Cruz, Heidelberg, Germany).

### BiFC, Immunofluorescence, and Laser Scanning Microscopy

For Bimolecular fluorescence complementation assays (BiFC; [35], [36]), cells were grown and transfected in eight-well chamber slides (NUNC, Roskilde, Denmark). Cells were imaged 24 h after transfection using a Zeiss LSM 510 Meta in confocal multitracking mode, with a x100/1.4-oil Apochromat objective (Zeiss, Jena, Germany) to generate 0.5 µm optical sections. Images were analyzed using ImageJ (Rasband, W.S., ImageJ, U. S. National Institutes of Health, Bethesda, Maryland, USA, http://imagej.nih.gov/ij/1997-2011). The number of transfected cells showing Venus or YFP complementation was quantified. As an index of the complementation efficiency, the Venus or YFP fluorescence intensity was measured in individual nuclei, and normalized to the mCherry fluorescence intensity within the same cells. At least 100 cells were measured per condition. Linear brightness and contrast adjustments were made for illustration purposes, but only after the analysis had been made.

Immunofluorescence analysis was performed on cells grown and transfected (when indicated) on coverslips, fixed for 10 min in 10% formalin and permeabilized for 10 min in 0.5% Triton X-100 in PBS. The primary antibodies were a rat anti-HA antibody (Roche), a rabbit anti-c-Fos antibody (sc-52; Santa Cruz) and a goat anti-TRAP220 (Med1) antibody (sc-5334; Santa Cruz). Secondary antibodies were anti-rat, anti-rabbit and anti-goat Alexa Fluor 488-conjugated antibodies (Invitrogen). When indicated, nuclei were counterstained with DRAQ5 (Biostatus Ltd., Shepshed, UK). The transfected or immunofluorescently stained array cells were imaged by confocal microscopy (see above). The entire nucleus was scanned to generate 0.2 µm optical sections. When detected, the array was typically present in 2 to 3 consecutive sections.

### Peptide SPOT analysis

Peptide SPOT synthesis [43] was performed essentially as described in [44]. Briefly, cellulose membrane-bound peptides were prepared in an automated Spot synthesizer (MultiPep, Intavis AG Bioanalytical Instruments, Köln, Germany) using Fmoc derivatives of amino-acids (Novabiochem, Darmstadt, Germany). GST-nTRIP6 was expressed in Escherichia coli BL21 and purified using glutathione Sepharose 4B beads (GE Healthcare, Freiburg, Germany). After activation of the membranes with methanol the membrane-bound peptide arrays were blocked for 3 h in blocking buffer (2% milk powder and 5% sucrose in Tris-buffered saline (TBS), pH 8.0) and then incubated overnight at 4°C with 10 µg/ml purified GST-nTRIP6 in blocking buffer, which was then detected with an anti-GST antibody (G1160; Sigma-Aldrich, München, Germany), revealed by a horseradish peroxidase conjugated anti-mouse antibody (Sigma-Aldrich) and ECL. The QRALAKDLIVPRRP peptide, recognized by the anti-GST antibody, was used as a positive control.

### Reverse transcription and real-time PCR

Total RNA was extracted using PeqGOLD TriFast (Peqlab Biotechnologie, Erlangen, Germany) and reverse-transcribed into cDNA. The mRNAs for MMP1, MMP13 and the ribosomal subunit 36B4 gene used for normalization, were quantified by real-time PCR using the ABI Prism Sequence Detection System 7000 (Applied Biosystems, Foster City, CA). The primers (Invitrogen) were as follows (5′ to 3′): MMP1: TGCTCATGCTTTTCAACCAGG and TGAGCCGCAACACGATGTAA; MMP13: GGCTGGAACCACATGGAAGAA and AGCAGATGGACCCCATGTTTG; 36B4: GGACCCGAGAAGACCTCCTT and GCACATCACTCAGAATTTCAATGG.

### Chromatin immunoprecipitation (ChIP)

ChIP assays were performed using the ChIP-IT Express kit (Active Motif, Rixensart, Belgium), following the manufacturer's instructions. Antibodies used were: anti-THRAP3 (Novus Biologicals, Cambridge, United Kingdom), anti-c-Fos (sc52; Santa Cruz) and anti-TRAP220 (Med1) (sc-5334; Santa Cruz). The isotype control antibodies were purchased from Diagenode (Liège, Belgium). Enrichment of the promoter of the MMP13 gene was determined by real-time PCR using the primers GTCGCCACGTAAGCATGTT and CTGTTGTCTTTCCGCAGAGA, and calculated as fold enrichment above background (isotype control antibody) after normalization to the input (ΔΔCt method).

### Statistical analysis

Where indicated, significant differences were assessed by t-test analysis, with values of *P*<0.05 sufficient to reject the null hypothesis.

## Results

### nTRIP6 dimerizes through its N-terminal pre-LIM region

**Figure 1:**
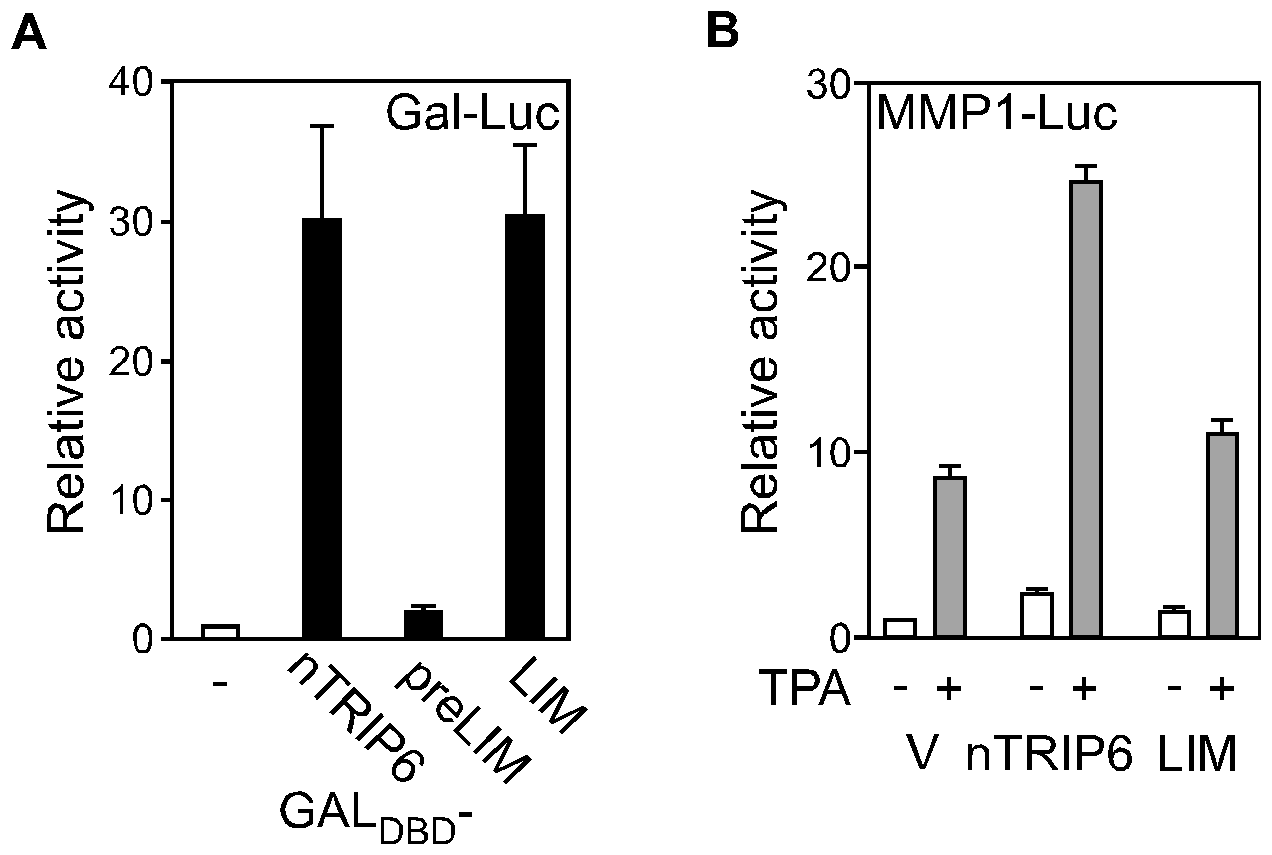
The N-terminus of nTRIP6 is required for its co-activator function. (**A**) HEK293 cells were cotransfected with a GAL-Luc reporter construct and Ubi-Renilla, together with expression vectors for either GAL_DBD_ or GAL_DBD_ fusions of nTRIP6, of nTRIP6 N-terminal pre-LIM region (preLIM) or of only the 3 LIM domains (LIM). Normalized luciferase activities are plotted relative to the activity obtained with GAL_DBD_ (mean ± SD of one representative experiment performed in triplicates). (**B**) HEK293 cells were cotransfected with a luciferase reporter gene driven by the AP-1-dependent MMP1 promoter (MMP1-Luc) and Ubi-Renilla, together with either an empty vector (V), an expression vector for nTRIP6 or for only the 3 LIM domains (LIM). Cells were treated with TPA as indicated. Normalized luciferase activities are plotted relative to untreated vector control (mean ± SD of one representative experiment performed in triplicates).

We have previously reported that nTRIP6 acts as a co-activator for AP-1, NF-κB and GR [19]–[21]. However, nTRIP6 does not harbour any functional domain known from other classical co-activators. The only domains in nTRIP6 which could account for a co-activator function are the three C-terminal LIM domains, functioning as protein-protein interaction modules. The logical hypothesis is therefore that the LIM domains are responsible for the co-activator function. To directly address this hypothesis, we made use of our prior observation that nTRIP6 co-activator function is transferable [19], i.e. that a fusion of nTRIP6 to GAL4 DNA binding domain (GAL4_DBD_) activates the expression of a reporter gene driven by GAL4-UAS (Fig. 1A). In this assay, the LIM domains alone fused to GAL4_DBD_ were sufficient to activate the reporter gene. The level of activation was similar to that achieved by a fusion of GAL4_DBD_ with the full-length nTRIP6 (GAL4_DBD_-nTRIP6). Fusion of GAL4_DBD_ with an nTRIP6 construct lacking the LIM domains (preLIM) had no effect on reporter gene expression (Fig. 1A). These results suggest that, as expected, nTRIP6 exert its co-activator function via its LIM domains. We thus studied whether the LIM domains are sufficient to co-activate AP-1 in reporter gene assays. Overexpression of nTRIP6 increased the responsiveness of the AP-1-dependent MMP1 (collagenase I) promoter (MMP1-Luc) to the phorbol ester TPA. However, overexpression of the LIM domains alone had no effect on the expression of the reporter gene (Fig. 1B). This result shows that the LIM domains lack a property needed for co-activation on the more complex transcription factor-bound promoter.

**Figure 2:**
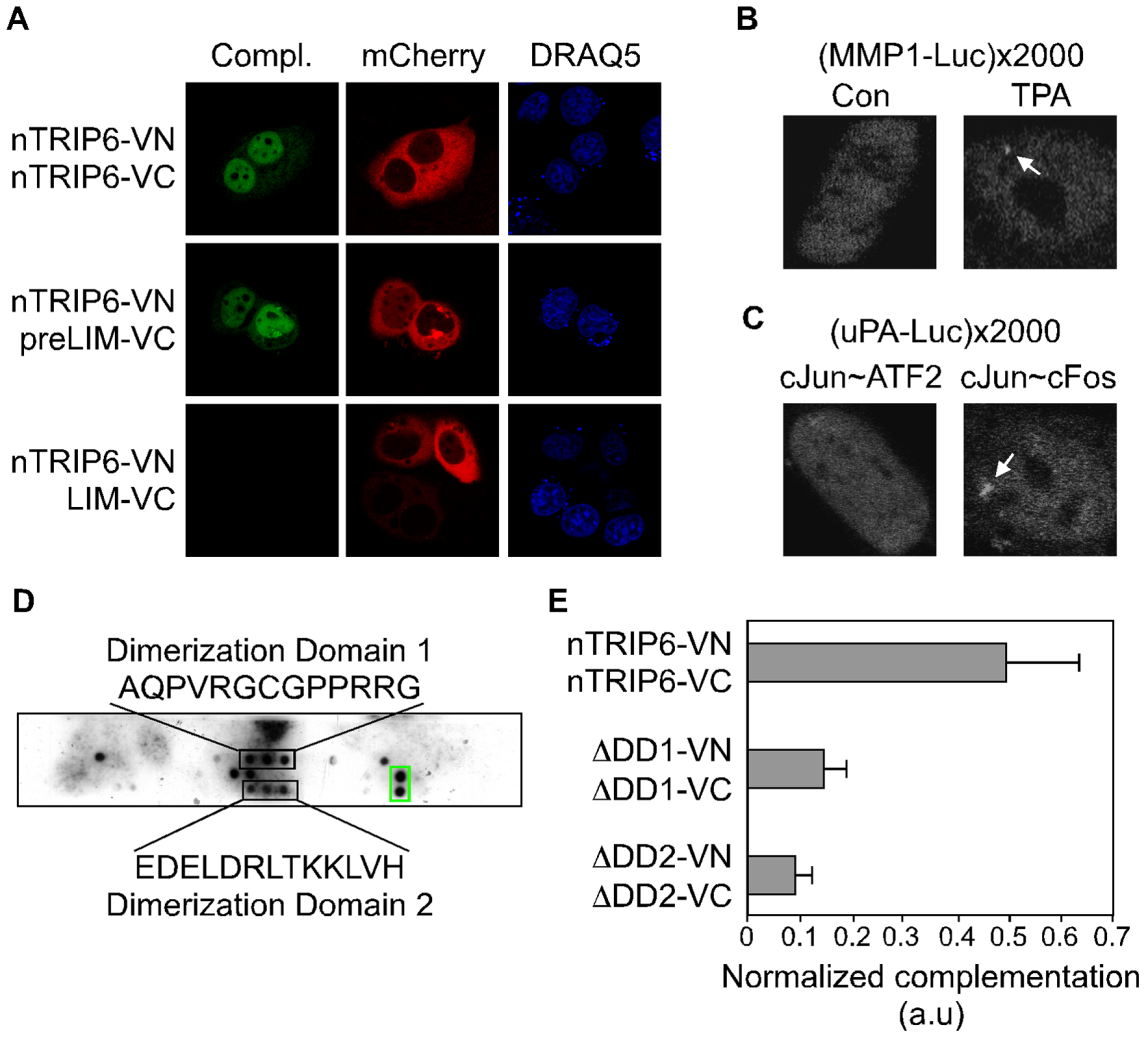
nTRIP6 dimerizes through discrete domains of its pre-LIM region. (**A**) HeLa cells were cotransfected with an expression vector for nTRIP6 fused to the N-terminal part of the Venus fluorescent protein (VN), and with an expression vector for either nTRIP6, nTRIP6 N-terminal pre-LIM region (preLIM) or only the 3 LIM domains (LIM) fused to the C-terminal part of Venus (VC), together with mCherry fused to a nuclear export signal (NES) as a transfection control. Cells were counterstained with DRAQ5 and imaged by confocal microscopy. The Venus fluorescence complementation (Compl.) was observed in 80 to 90% of the cells transfected with nTRIP6-VN and nTRIP6-VC or preLIM-VC, but in none of the cells transfected with nTRIP6-VN and LIM-VC. (**B, C**) nTRIP6 dimerizes at the promoter of AP-1 target genes. NIH-3T3 fibroblast cell lines containing an integrated array of multiple copies of the indicated reporter gene were cotransfected with nTRIP6-VN and nTRIP6-VC. Cells were treated for 3 h with TPA (**B**) or cotransfected with an expression vector for either the single chain AP-1 c-Jun∼ATF2 or the single chain AP-1 c-Jun∼c-Fos (**C**). Cells were imaged by confocal microscopy. Nuclei of representative cells are shown. A selective enrichment of the Venus complementation to the array (arrow) was observed in 70 to 80% of the TPA-treated cells (**B**) and the c-Jun∼c-Fos-transfected cells showing complementation (**C**). (**D**) Mapping of two nTRIP6 dimerization domains. Peptides spanning the entire sequence of nTRIP6 pre-LIM region were synthesized on a cellulose membrane as 15 mers, each shifted by 3 amino-acids. The membrane was incubated with recombinant nTRIP6 fused to GST, which was then detected by an anti-GST antibody. The two positive spots boxed in green correspond to a control peptide recognized by the anti-GST antibody. (**E**) Both domains are required for nTRIP6 dimerization. HeLa cells were cotransfected with the indicated combination of expression vectors for nTRIP6, nTRIP6 lacking the dimerization domain 1 (ΔDD1) or nTRIP6 lacking the dimerization domain 2 (ΔDD2), fused to either VN or VC, together with the mCherry-NES expression vector as a transfection control. Venus complementation was quantified by measuring the Venus fluorescence intensity in individual nuclei, normalized to the mCherry fluorescence intensity within the same cells, and is presented as arbitrary units (a.u.; mean ± SD of three independent experiments). Representative images are shown in Fig. S3C.

What could be the contribution of the pre-LIM region to the AP-1 co-activation? To reconcile these apparently contradictory results, we hypothesized that nTRIP6 homodimerizes through its pre-LIM region, and that this dimerization is essential for the co-activator function. We tested whether nTRIP6 interacts with itself in living cells using bimolecular fluorescence complementation of the Venus fluorescent protein (BiFC; [35]). Venus complementation was indeed observed in the nucleus of 80 to 90% of the cells co-transfected with nTRIP6 fused to the N-terminus of Venus (nTRIP6-VN) and nTRIP6 fused to the C-terminus of Venus (nTRIP6-VC) (Fig. 2A). The LIM domains of nTRIP6 were dispensable for this interaction, as indicated by the complementation between nTRIP6-VN and preLIM-VC (nTRIP6 N-terminal pre-LIM region fused to VC; Fig. 2A). Furthermore, no complementation was observed between nTRIP6-VN and the LIM domains alone fused to VC (LIM-VC), although the cells were efficiently transfected, as indicated by the cytosolic expression of the red fluorescent protein mCherry fused to a nuclear export sequence used as a transfection control (Fig. 2A). All VC and VN fusion constructs were expressed and localized to the nucleus (Fig. S1). Thus, nTRIP6 interacts with itself through its pre-LIM region. Although we do not know about the stoichiometry of this interaction, homodimerization or higher order complexes, we refer to it as homodimerization for clarity.

If this dimerization were indeed required for the co-activator function of nTRIP6, it should occur at the promoter of AP-1 dependent genes. To visualize the promoter-associated homodimerization of nTRIP6, we generated a reporter cell line containing an integrated array of multiple copies of the AP-1-dependent MMP1-Luc reporter gene. The array was generated as previously described [20], [21], by transfecting the reporter plasmid together with the pΔBN-AR1 plasmid [39], which initiates events similar to gene amplification in cancer cells, leading to tandem repeats of up to 10 000 copies [39], [45]. The high local concentration of binding sites on the amplified gene array permits the visualization of the binding of fluorescently tagged proteins, as previously shown by us and others for NF-κB-, AP-1- and GR-dependent array cell lines [20], [21], [42]. Using this method we generated NIH-3T3 fibroblasts carrying several hundred integrated MMP1-Luc plasmid copies in discrete loci (Fig. S2). One of the cell clones, clone 12c, was estimated by real-time PCR to have an integrated array of about 2000 gene units. The presence of the MMP1-Luc array was confirmed by DNA fluorescent in situ hybridization using a fragment of the luciferase coding sequence as a probe. The staining was restricted to a single spot within the nucleus of the 12c cells, and was seen in 100% of the cells, whereas only background staining was visible in the parental NIH-3T3 cells (Fig. S2B). TPA treatment induced luciferase activity in clone 12c (Fig. S2C), suggesting that AP-1 was able to bind to its cognate response elements on the array and to activate transcription from the MMP1-Luc genes. To confirm the functionality of the array, we studied the recruitment of c-Fos, nTRIP6, the Mediator complex protein Med1/TRAP220, and RNA polymerase II (Pol II; Fig. S2D, E). Transfected nTRIP6 fused to YFP was located in the nucleus, and was recruited to the array upon TPA treatment, as shown by the enrichment to a single bright spot in the nucleus. Similarly, Pol II fused to GFP, as well as endogenous c-Fos and TRAP220/Med1, both detected by immunofluorescence, were recruited to the array upon TPA treatment (Fig. S2D, E). This specific enrichment to the array was observed in 70 to 80% of the transfected cells. These results confirm that the MMP1-Luc array is functional and responds to TPA treatment, and encouraged us to test whether nTRIP6 homodimerizes on the promoter of an AP-1 target gene, by performing BiFC assays in this array cell line. Venus fluorescence complementation was observed in the nucleus of the cells co-transfected with nTRIP6-VN and nTRIP6-VC. The fluorescence complementation was enriched to the array upon TPA treatment (Fig. 2B), suggesting that indeed nTRIP6 homodimers are recruited to the AP-1-bound promoter. To confirm that the nTRIP6 homodimer is selectively recruited to the promoter via an interaction with AP-1, we made use of a previously established reporter cell line, which harbours an array of a reporter gene driven by the minimal AP-1-dependent enhancer of the urokinase type plasminogen activator (uPA) gene (-1977/-1858uPA-TATA-Luc). The uPA gene enhancer [46], [47] harbours response elements for both the AP-1 complexes cJun:c-Fos which interacts with nTRIP6, and for c-Jun:ATF2 which does not interact with nTRIP6 [20]. To detect specific AP-1 complex-dependent recruitment of the nTRIP6 homodimer, these array cells were co-transfected with both nTRIP6 BiFC constructs, together with “single chain” AP-1 constructs, in which the coding sequences of either c-Jun and c-Fos, or c-Jun and ATF2 were fused in frame using a flexible linker [37]. In the presence of the c-Jun:ATF2 single chain AP-1, which does not interact with nTRIP6, nTRIP6 homodimers were homogenously distributed in the nucleus. However, in cells co-transfected with the c-Jun:c-Fos construct, which does interact with nTRIP6, nTRIP6 homodimers were enriched to the array (Fig. 2C). Given that nTRIP6 is tethered to AP-1-bound promoters through a direct interaction with c-Fos [20], these results strongly suggest that the nTRIP6 homodimer is specifically recruited to the promoter via its interaction with AP-1.

Our results show that the pre-LIM region of nTRIP6 mediates homodimerization. To further map the dimerization site, we performed a peptide SPOT analysis [43]. Overlapping peptides covering the pre-LIM region of nTRIP6 were synthesized on nitrocellulose membranes, which were then probed with recombinant nTRIP6 fused to GST (Fig. 2D). This screen identified 2 peptides able to interact with nTRIP6, AQPVRGCGPPRRG and EDELDRLTKKLVH, corresponding to amino acid positions 175-187 and 253-265, respectively. We then tested the involvement of these 2 domains, named Dimerization Domain (DD)1 and DD2 respectively, in the homodimerization of nTRIP6 using the BiFC assay (Fig. 2E). The complementation between the VC and VN fusions of nTRIP6 lacking DD1, as well as the complementation of the constructs lacking DD2, was strongly reduced compared to the complementation observed with the wild type constructs. The wild type and deletion constructs fused to VC and VN were expressed at similar levels and located in the nucleus (Fig. S3). This result indicates that both domains are required for the optimal homodimerization of nTRIP6.

### nTRIP6 homodimerization is required for its co-activator function

**Figure 3:**
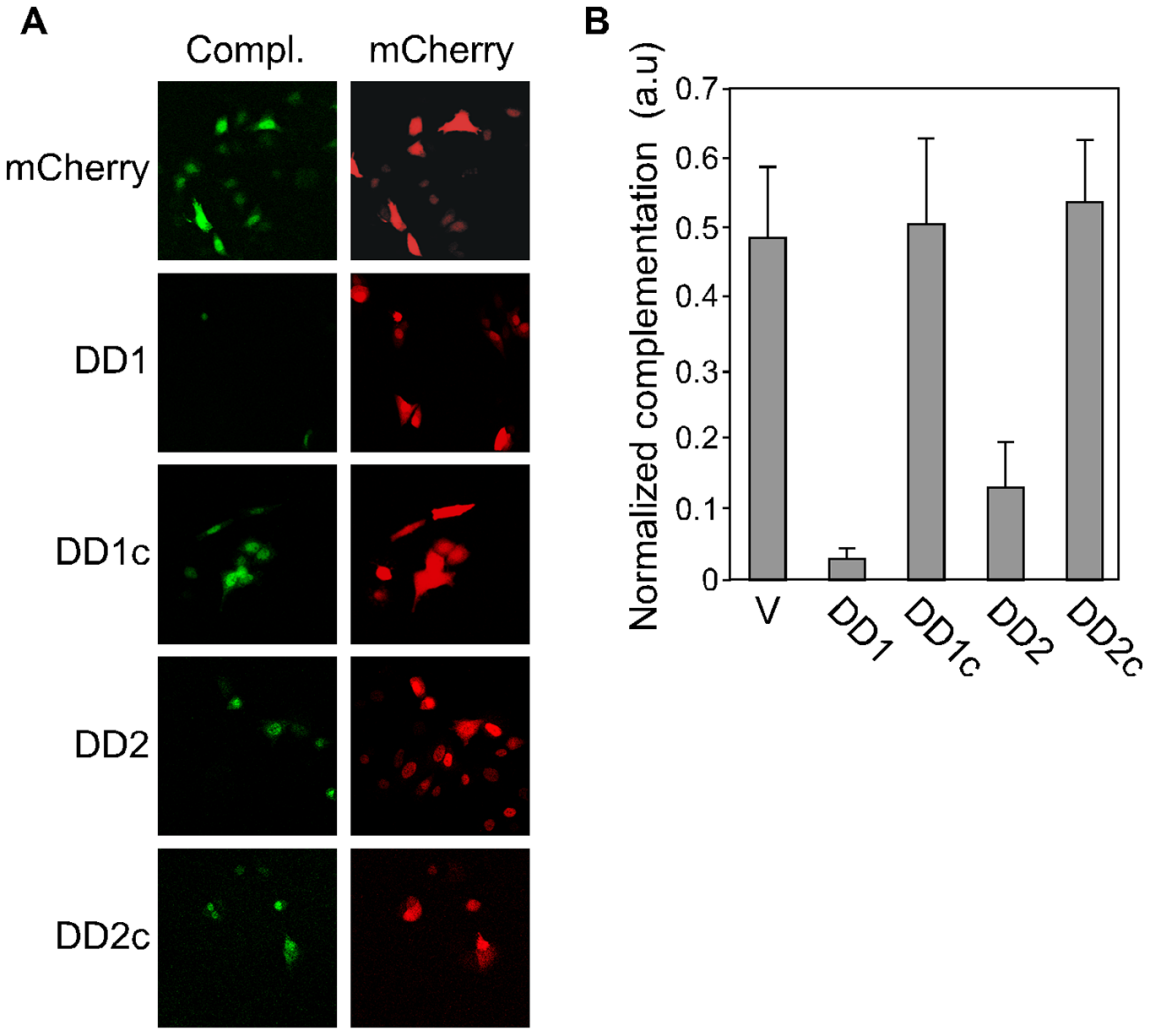
Blocking peptides inhibit nTRIP6 dimerization. HeLa cells were cotransfected with expression vectors for nTRIP6 fused to the N-terminal part of Venus and for nTRIP6 fused to the C-terminal part of Venus, together with expression vectors for either mCherry fused to a nuclear localization signal (NLS), a peptide corresponding to the sequence of the dimerization domain 1 fused to an NLS and to mCherry (DD1), a scrambled version of the DD1 peptide (DD1c), a peptide corresponding to the sequence of the dimerization domain 2 fused to an NLS and to mCherry (DD2), or a scrambled version of the DD2 peptide (DD2c). (**A**) Venus complementation (Compl.) was imaged by confocal microscopy and representative cells are shown. (**B**) Complementation was quantified by measuring the Venus fluorescence intensity in individual nuclei, normalized to the mCherry fluorescence intensity within the same cells, and is presented as arbitrary units (a.u.; mean ± SD of three independent experiments).

**Figure 4:**
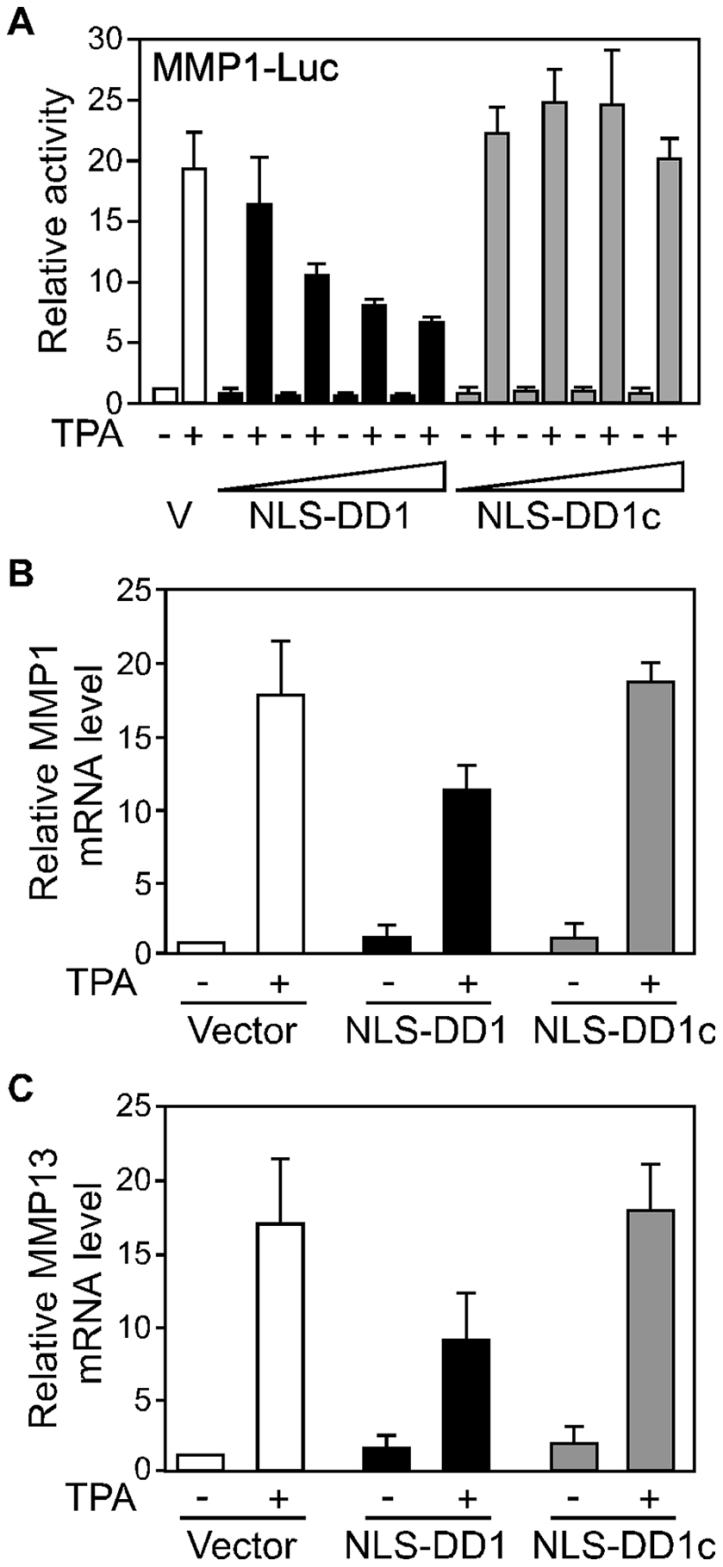
The DD1 peptide inhibits AP-1. (**A**) HEK293 cells were cotransfected with the AP-1-dependent MMP1-Luc reporter construct and Ubi-Renilla, together with either a control empty vector (V) or increasing amounts of an expression vector for the mCherry-NLS fusion of the DD1 peptide or its scrambled version (DD1c). Cells were treated with TPA as indicated. Normalized luciferase activities are plotted relative to untreated vector control (mean ± SD of one representative experiment performed in triplicates). (**B, C**) HEK293 cells were transfected with either a control empty vector, an expression vector for the mCherry-NLS fusion of the DD1 peptide or its scrambled version (DD1c). Cells were treated with TPA as indicated. The relative levels of MMP1 (**B**) and MMP13 (**C**) mRNAs were determined by reverse transcription and real-time PCR, and are plotted relative to the untreated vector control (mean ± S.D. of three independent experiments).

In order to now address the functional relevance of nTRIP6 homodimerization, we designed short peptides corresponding to the 13 amino acid sequences of the dimerization domains, with the prediction that they should competitively block dimerization. The peptides were fused to a nuclear localization signal (NLS) to prevent any interference with a putative dimerization of TRIP6 in the cytosol, and to mCherry to trace their expression and localization. Both DD1 and DD2 peptides inhibited nTRIP6 homodimerization in the BiFC assay, whereas scrambled versions of the peptides used as controls, had no effect (Fig. 3A). Quantification of the complementation showed that the DD1 peptide was more efficient than the DD2 peptide (Fig. 3B). Furthermore, sequence alignment of the corresponding pre-LIM regions of related LIM domain proteins of the zyxin family showed high conservation of the DD2 sequence, but not of the DD1 sequence (Fig. S4). Thus, the DD2 peptide might also affect the function of other LIM domain proteins. For these reasons, only the DD1 peptide was used for further experiments. We first used it to study whether nTRIP6 homodimerization is required for its co-activator function in reporter gene assays (Fig. 4A). The peptide dose-dependently reduced the induction by TPA of the MMP1-Luc reporter gene, whereas the scrambled control peptide had no effect (Fig. 4A). Importantly, the DD1 peptide did not interfere with the interaction between AP-1 and nTRIP6 (Fig. S5). As an additional specificity control, the DD1 and the scrambled control peptides were fused to a nuclear export signal (NES) instead of an NLS. The NES-DD1 peptide had no effect on the induction of the MMP1-Luc reporter gene (Fig. S6). We then studied the effect of the peptide on the induction of endogenous AP-1-regulated genes. In HEK293 cells, overexpression of the DD1 peptide repressed the induction by TPA of the AP-1 target genes MMP-1 and MMP13, whereas the control scrambled peptide had no effect (Fig. 4B, C).

### nTRIP6 homodimers mediate the promoter recruitment of THRAP3 and of the Mediator complex

**Figure 5:**
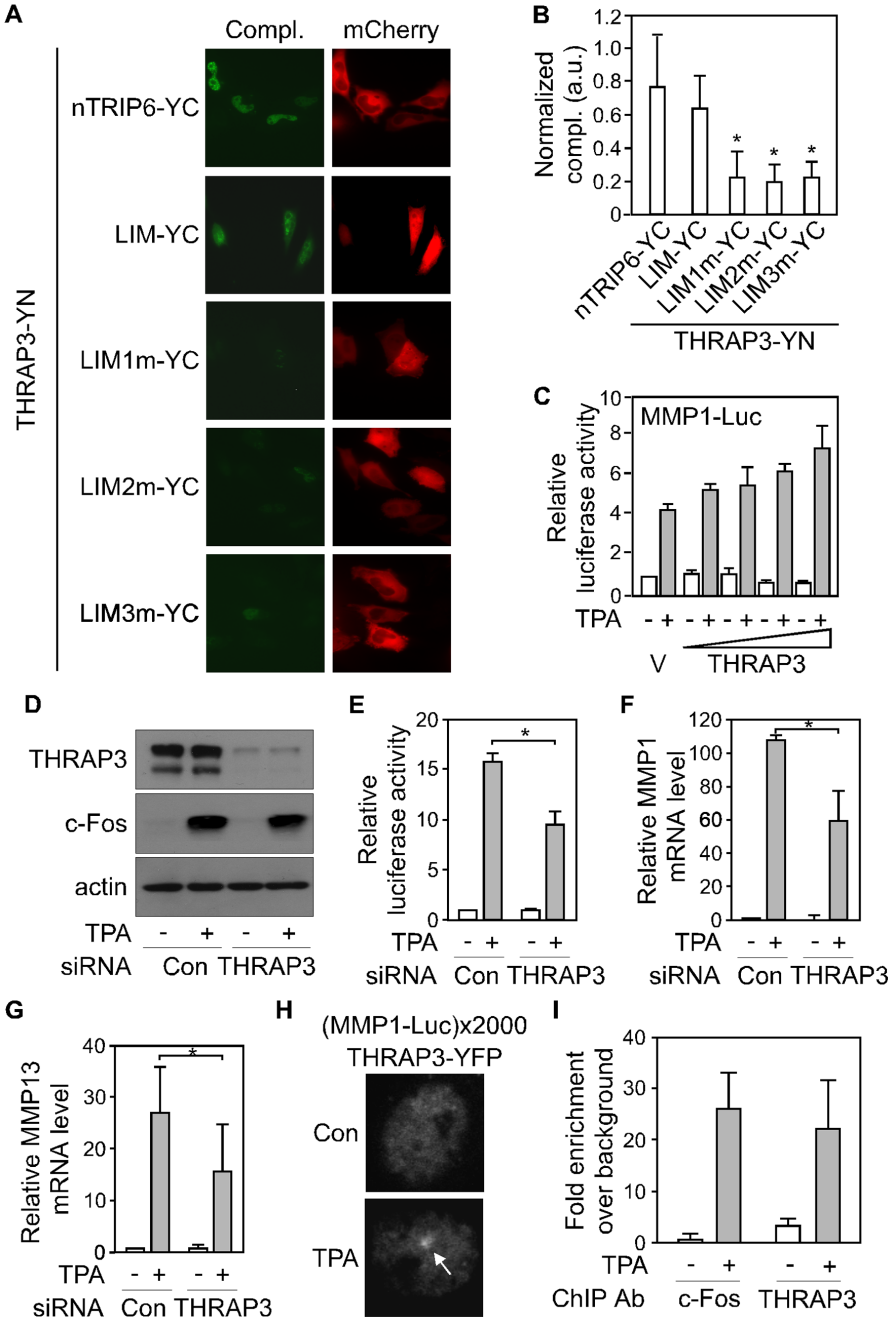
THRAP3 is a co-activator for AP-1. (**A**) THRAP3 interacts with nTRIP6. HEK293 cells were cotransfected with THRAP3 fused to the N-terminal part of YFP (YN) together with C-terminal part of YFP (YC) fusions of either nTRIP6, only the 3 LIM domains (LIM), or nTRIP6 mutants in which two of the coordinating cysteines in the two zinc fingers of the LIM domain 1 (LIM1m), of the LIM domain 2 (LIM2m) or of the LIM domain 3 (LIM3m) were mutated to alanines. The cells were cotransfected with mCherry fused to a nuclear export signal (NES) as a transfection control. Cells were imaged by confocal microscopy and representative images are shown. (B) The relative YFP complementation (Compl.) was quantified by measuring the YFP fluorescence intensity in individual nuclei, normalized to the mCherry fluorescence intensity within the same cells, and is presented in arbitrary units (a.u.; mean ± SD of three independent experiments; *, *P*<0.05). (**C**) Cos7 cells were cotransfected with the AP-1-dependent MMP1-Luc reporter construct, together with either a control empty vector (V) or increasing amounts of an expression vector for THRAP3. Cells were treated with TPA as indicated. Luciferase activities are plotted relative to untreated vector control (mean ± SD of one representative experiment performed in triplicates). (**D–G**) HEK293 cells were transfected with either a control siRNA (Con) or an siRNA targeting THRAP3, together with the MMP1-Luc reporter construct and Ubi-Renilla (**E**). Cells were treated with TPA as indicated. (**D**) Cell lysates were subjected to Western Blotting using antibodies against THRAP3, c-Fos and actin as a loading control. (**E**) Normalized luciferase activities are plotted relative to the values obtained with untreated control siRNA transfected cells (mean ± SD of one representative experiment performed in triplicates; *, *P*<0.05). (**F**, **G**) The relative levels of MMP1 (**F**) and MMP13 (**G**) mRNAs were determined by reverse transcription and real-time PCR, and are plotted relative to the values obtained with untreated control siRNA transfected cells (mean ± S.D. of three independent experiments; *, *P*<0.05). (**H**) NIH-3T3 fibroblast cells containing an integrated array of multiple copies of the MMP1-Luc reporter gene were transfected with a YFP fusion of THRAP3, treated for 3 h with solvent alone (Con) or TPA and imaged by confocal microscopy. Nuclei of representative cells are shown. A selective enrichment of the YFP fluorescence to the array (arrow) was observed in 60 to 70% of the transfected, TPA-treated cells. (**I**) Chromatin immunoprecipitation (ChIP) was performed in HEK293 cells treated with TPA as indicated, using the indicated antibodies (Ab) or isotype control antibodies. Enrichments of the MMP13 gene promoter were determined by real-time PCR, and plotted as fold enrichment above background (isotype control antibody) after normalization to the input (mean ± SD of three independent experiments).

Together, our results show that nTRIP6 dimerizes via discrete domains within its pre-LIM region, in order to exert its co-activator function through its LIM domains. Given that LIM domains are not co-activator domains *per se* but protein-protein interaction modules, the logical hypothesis is that nTRIP6 homodimers mediate the recruitment of other co-activator or co-activator complexes to the transcription factor-bound promoter. In a proteomics-based large-scale study of protein-protein interactions [48], we identified TRIP6 as interacting with the thyroid hormone receptor–associated protein 3 (THRAP3 or TRAP150), a subunit of the Mediator complex [49]. We therefore tested whether nTRIP6 interacts with THRAP3 in the nucleus of HeLa cells using the BiFC assay (Fig. 5A, B). YFP fluorescence complementation indeed documented this interaction. It was observed in the nucleus of 80 to 90% of the cells co-transfected with nTRIP6 fused to the C-terminal half of YFP (YC) together with THRAP3 fused to the N-terminal half of YFP (YN). Furthermore, a similar complementation was observed in cells co-transfected with the LIM domains alone fused to YC and THRAP3-YN, showing that the N-terminal pre-LIM region is dispensable for this interaction. To study the contribution of the individual LIM domains of nTRIP6 to its interaction with THRAP3, we mutated in the BiFC construct the coordinating cysteines of the two zinc fingers of each LIM domain to alanines. Cells co-transfected with THRAP3-YN together with either of the three nTRIP6 LIM mutants fused to YC showed significantly reduced complementation as compared to the wild type nTRIP6 construct (Fig. 5A, B), suggesting that all three LIM domains participate in the interaction.

**Figure 6:**
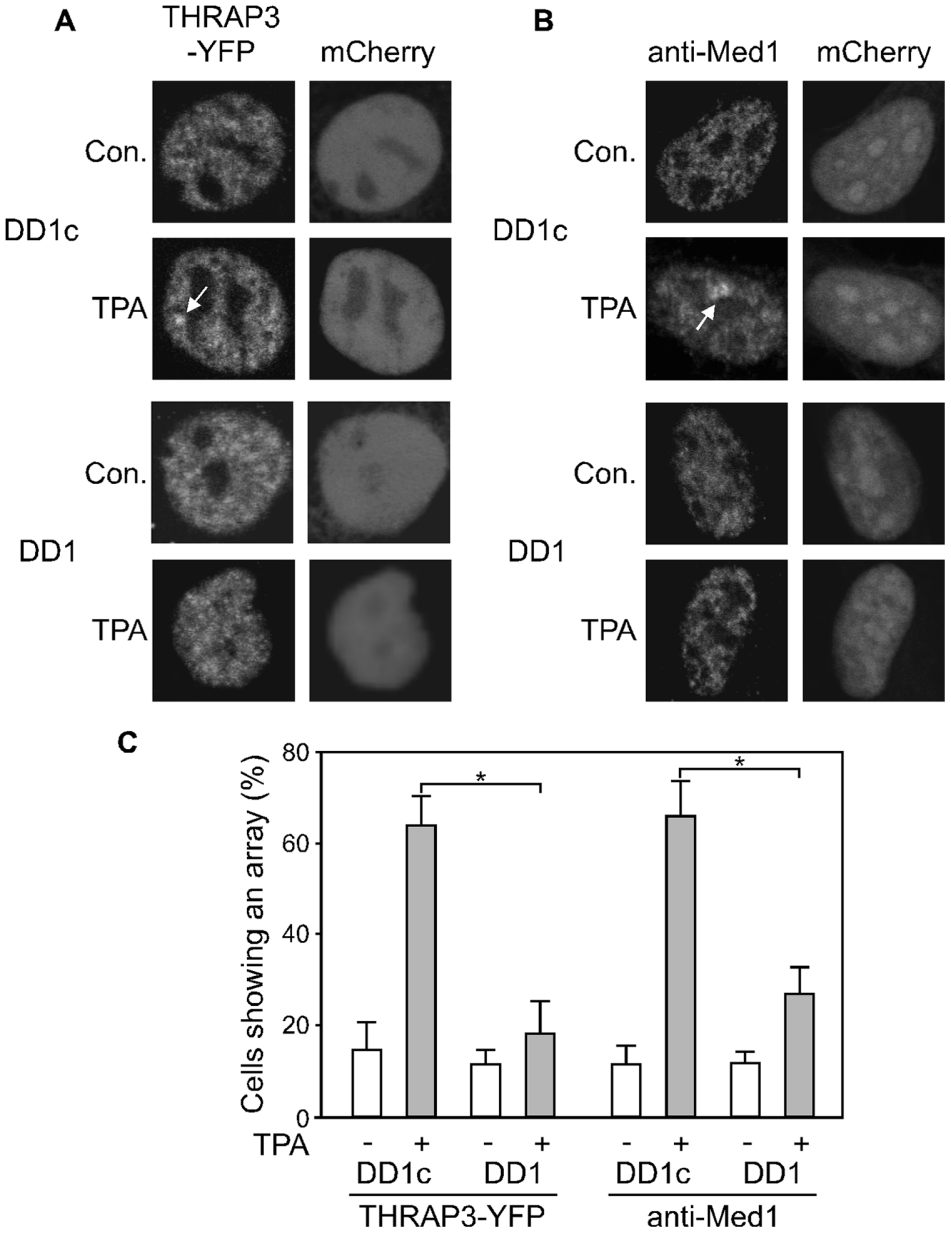
nTRIP6 homodimerization is required for the promoter recruitment of THRAP3 and of the Mediator complex. The MMP1-Luc array cells were co-transfected with THRAP3 fused to YFP (**A**) or an empty vector (**B**), together with either the mCherry-NLS fusion of the DD1 peptide or its scrambled version (DD1c). Cells were treated with solvent alone (Con.) or TPA for 3 h as indicated. Empty vector transfected cells were subjected to immunofluorescent labelling using an anti-Med1/TRAP220 antibody (**B**). Cells were imaged by confocal microscopy, and nuclei of representative cells are shown. The arrow depicts the enrichment of the fluorescence to the array. (**C**) The number of cells in which the array was visible was counted, and is presented as percent of the transfected cells (mean ± SD of three independent experiments; *, *P*<0.05).

Is the nTRIP6-THRAP3 interaction relevant for the activation of AP-1 dependent transcription? To tackle this question, we first tested whether THRAP3 acts on AP-1-regulated promoters. In Cos7 cells, overexpression of THRAP3 dose-dependently increased the induction of the AP-1-dependent reporter gene by TPA (Fig. 5C). Conversely, silencing THRAP3 by siRNA inhibited the induction of the reporter gene (Fig. 5E) and of the endogenous AP-1 target genes MMP1 and MMP13 by TPA (Fig. 5F, G), while it had no effect on the induction of c-Fos by TPA (Fig. 5D). We then used the MMP1-Luc array cells to study the promoter recruitment of THRAP3. A transfected YFP fusion of THRAP3 was located in the nucleus, and was recruited to the array upon TPA treatment, as shown by the enrichment to a single bright spot in the nucleus (Fig. 5H). These results were confirmed by chromatin immunoprecipitation (ChIP) experiments, which showed that endogenous THRAP3 was recruited to the MMP13 promoter upon induction (Fig. 5I). Thus, THRAP3 is a co-activator for AP-1. Given the interaction of THRAP3 with nTRIP6, the co-activator function of THRAP3 should depend on nTRIP6 homodimerization. We again used the array cells to test this hypothesis. Co-transfection of the DD1 peptide, which blocks nTRIP6 dimerization, significantly reduced the recruitment of THRAP3-YFP to the array, as compared to the scrambled control peptide (Fig. 6A, C). This result strongly suggests that the promoter recruitment of THRAP3 depends on nTRIP6 homodimerization. Since THRAP3 is a subunit of the Mediator complex, the promoter recruitment of other Mediator complex components might also require nTRIP6 homodimerization. Indeed, the TPA-induced recruitment of a core Mediator complex subunit, Med1/TRAP220, was inhibited by the DD1 peptide, as assessed by immunofluorescence analysis of the array cells (Fig. 6B, C). Together, these results show that THRAP3 is an nTRIP6 homodimer-dependent AP-1 co-activator, and strongly suggest that nTRIP6 mediates the recruitment of the Mediator complex to AP-1-bound promoters via an interaction with THRAP3.

### GR inhibits the recruitment of THRAP3 to AP-1-regulated promoters

**Figure 7:**
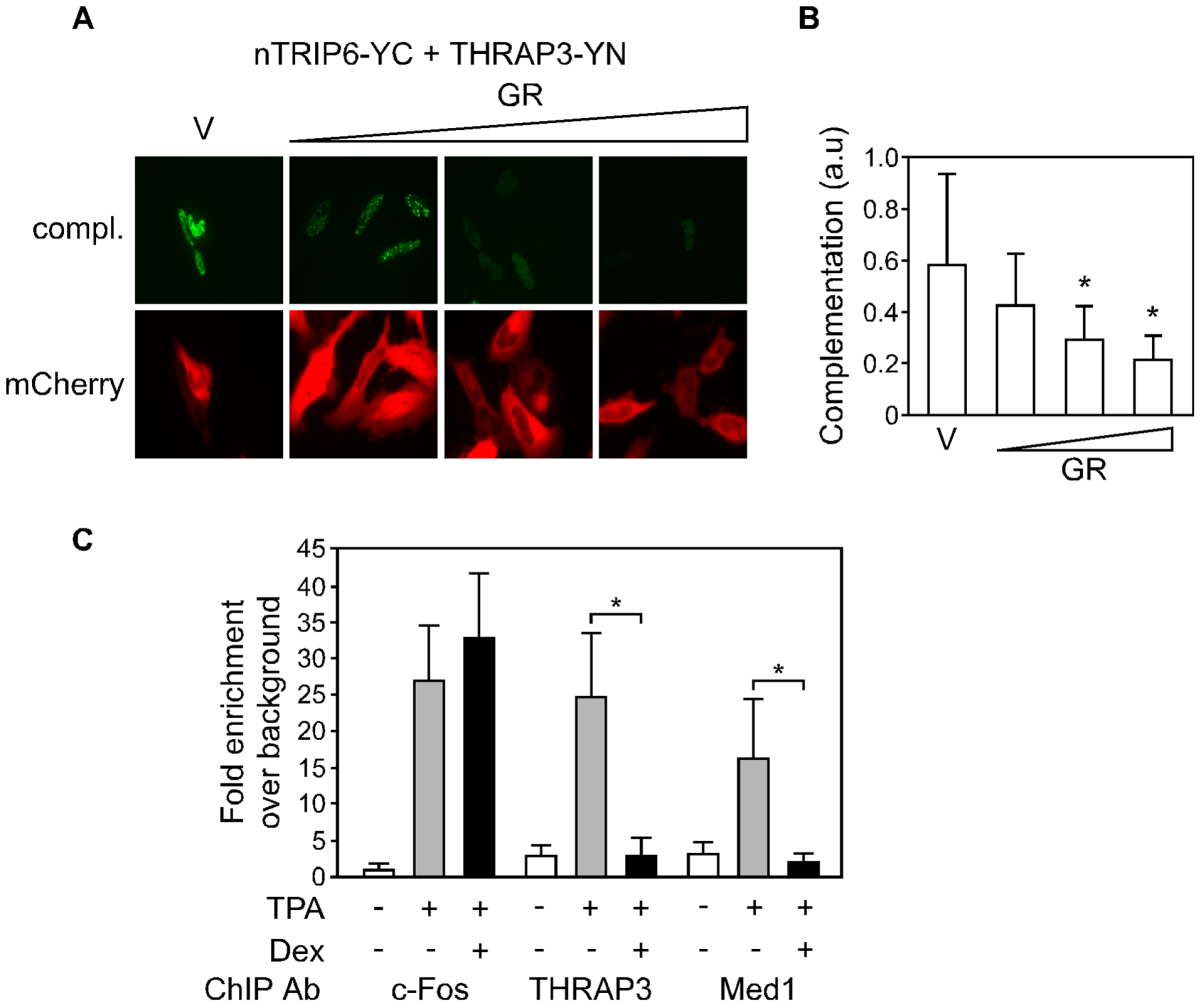
GR prevents the recruitment of THRAP3. (**A, B**) HEK293 cells were cotransfected with nTRIP6 fused to the C-terminal part of YFP (YC) and THRAP3 fused to the N-terminal part of YFP (YN), together with either a control empty vector (V) or increasing amounts of an expression vector for GR, and mCherry fused to a nuclear export signal (NES) as a transfection control. Cells were imaged by confocal microscopy. (**A**) Images of representative cells are shown. (**B**) YFP complementation was quantified by measuring the YFP fluorescence intensity in individual nuclei, normalized to the mCherry fluorescence intensity within the same cells, and is presented in arbitrary units (a.u.; mean ± SD of three independent experiments; *, *P*<0.05). (**C**) HEK293 cells were treated TPA in the presence or absence of dexamethasone (Dex) as indicated, and chromatin immunoprecipitation was performed on the MMP13 promoter using the indicated antibodies (Ab) or isotype control antibodies. Enrichments of the MMP13 gene promoter were determined by real-time PCR, and plotted as fold enrichment above background (isotype control antibody) after normalization to the input (mean ± SD of three independent experiments; *, *P*<0.05).

We have previously reported that nTRIP6 is also involved in the transrepression of AP-1 by GR [19], [20]. In this mode of repression by GR, the interaction between nTrip6 and c-Fos in not disrupted, and nTrip6 recruitment to the c-Fos bound promoter is not prevented. Rather, one important function of nTrip6 in transrepression is to mediate the tethering of GR to the promoter-bound AP-1 [19], [20]. Given that nTRIP6 dimerization is essential for its co-activator function, we tested whether GR inhibits nTRIP6 dimerization. In the BiFC assay, the complementation between nTRIP6-VN and nTRIP6-VC was not affected by the dexamethasone-mediated activation of co-transfected mCherry-GR (Fig. S7). Thus, GR does not hamper nTRIP6 dimerization. The next logical hypothesis is that the tethering of GR interferes with the recruitment of nTRIP6-dependent co-activators. The LIM domains of nTRIP6 are required for the interaction with both THRAP3 and GR. Thus, we speculated that THRAP3 and GR might compete for the interaction with nTRIP6. In a BiFC assay, the complementation between nTRIP6-YC and THRAP3-YN was significantly reduced by overexpressed GR (Fig. 7A, B), showing that GR inhibits the interaction between nTRIP6 and THRAP3. We then studied by ChIP the effect of GR on THRAP3 recruitment to an AP-1-regulated promoter (Fig. 7C). As previously reported [29], dexamethasone treatment did not interfere with the recruitment of c-Fos to the MMP13 promoter. However, the TPA-induced recruitment of THRAP3 was strongly inhibited by dexamethasone. Similarly, dexamethasone treatment inhibited the TPA-induced recruitment of Med1/TRAP220 (Fig. 7C). Thus, GR represses the recruitment of THRAP3, an nTRIP6-dependent AP-1 co-activator.

## Discussion

We report here that (i) the transcriptional co-activator function of nTRIP6, the nuclear isoform of the LIM domain protein TRIP6, requires its homodimerization, (ii) nTRIP6 homodimers mediate the promoter recruitment of the Mediator complex component THRAP3, which thereby acts as a co-activator for AP-1, and (iii) GR represses AP-1 activity at least in part by preventing the nTRIP6-mediated recruitment of THRAP3.

### nTRIP6 acts as a homodimer

We had previously shown that nTRIP6 acts as a co-activator for several transcription factors, although it does not harbour any known co-activator domain. Given that nTRIP6 carries three protein interaction domains (LIM domains), it is plausible that it acts as an adaptor co-activator. The LIM domain would serve not only to interact with the promoter-bound transcription factor [19]–[21], but also for the assembly of other regulatory proteins. Surprisingly, our results show that not only the LIM domains, but also the N-terminal pre-LIM region of nTRIP6 participate in the co-activator function. An earlier analysis of TRIP6 transcriptional regulatory function had suggested that TRIP6 contains at least two “transactivation domains”, one located within the LIM domain region and the other within the pre-LIM region [50]. According to our results both regions exert essential yet different functions: discrete domains within the pre-LIM mediate nTRIP6 homodimerization, whereas the LIM domains are directly involved in transcriptional activation via the recruitment of co-activators, in particular the novel AP-1 co-activator THRAP3. The LIM domains alone covalently fused to a DNA binding domain (GAL4_DBD_) can activate transcription, showing that they are sufficient to interact with and recruit co-activators. Indeed, THRAP3 interacts with the LIM domains of nTRIP6 and its promoter recruitment depends on nTRIP6. Thus, nTRIP6 regulates transcription as an adaptor co-activator, similarly to LIM-only proteins. However, when nTRIP6 was recruited to the promoter via the interaction of its LIM domains with a promoter-bound transcription factor, the LIM domains were not sufficient to co-activate AP-1. The N-terminal pre-LIM region was essential for the co-activator function, by mediating the homodimerization of nTRIP6. Although, from the BiFC experiments, nTRIP6 interacts with itself, we cannot conclude as to the stoichiometry of the complex: nTRIP6 exists at least as a homodimer, but we cannot exclude the existence of higher order complexes. We identified two dimerization domains within the pre-LIM region of nTRIP6, one of which is conserved in other related LIM domain proteins of the Zyxin family. Thus, it is tempting to speculate that these other LIM domain proteins also dimerize, which might be important for their reported transcriptional co-regulator action [51]–[56]. Why is nTRIP6 homodimerization required for its co-activator function? nTRIP6 uses its LIM domains for interacting with both the transcription factors and the co-activator THRAP3. Thus, if nTRIP6 were recruited as a monomer to the promoter-bound transcription factor, then the LIM domains might not be accessible for an interaction with THRAP3, which appears to depend on all three LIM domains. In an nTRIP6 homodimer, one set of LIM domains would mediate the recruitment to the promoter-bound transcription factor, and the second set would be available to interact with and recruit other co-activators such as THRAP3. This scenario is corroborated by our observation that blocking nTRIP6 dimerization abolishes the promoter recruitment of THRAP3.

### THRAP3 is an nTRIP6-dependent AP-1 co-activator

Based on its ability to interact with nTRIP6, we have identified THRAP3 as a novel co-activator for AP-1: (i) overexpression of THRAP3 increased the transcriptional activity of AP-1, while (ii) silencing of THRAP3 reduced the expression of AP-1 target genes, and (iii) THRAP3 was indirectly recruited to the AP-1-bound promoter in an nTRIP6 homodimer dependent manner. THRAP3 was first identified as a component of the Mediator co-activator complex [49], and has since been shown to co-activate several transcription factors, such as the Peroxisome proliferator-activated receptor gamma [57] and the heterodimeric transcription factor circadian locomotor output cycles kaput (CLOCK)-brain, muscle Arnt-like 1 (BMAL1) [58]. The siRNA targeting THRAP3 did not totally abolish the induction of AP-1 target genes (Fig. 5F, G). However, the knockdown of THRAP3 was not total (Fig. 5D). A likely interpretation is that the expression of THRAP3 is not limiting for its co-activator function, and that the residual THRAP3 levels after silencing are sufficient to promote transcription. The fact that THRAP3 is expressed at relatively high levels is not entirely surprising considering the other functions of THRAP3, for example in splicing or in the DNA damage response [38], [59]. This might also explain why THRAP3 overexpression only moderately increased AP-1 activity (Fig. 5B).

THRAP3 most likely does not represent a core component of the Mediator complex [60]–[62]. Indeed, several types of functionally distinct Mediator complexes, which vary in their composition, have been described (reviewed in [63], [64]). The Mediator complex proteins MED1 and MED14 have been identified as interacting with the GR [65]. How the Mediator complex is recruited to AP-1 has remained elusive. Our results reveal that inhibiting the recruitment of THRAP3 by blocking nTRIP6 dimerization also inhibited the recruitment of another Mediator complex subunit (Med1/TRAP220). This is, to our knowledge, the first identification of the mechanism whereby the Mediator complex is recruited to AP-1-regulated promoters. Furthermore, this observation suggests that a THRAP3-containing Mediator complex subtype is indirectly recruited to AP-1 target genes through the interaction between THRAP3 and nTRIP6. Thus, the function of THRAP3 in AP-1 co-activation may be to recruit the Mediator complex, as is the case for THRAP3-mediated CLOCK/BMAL1 co-activation [58]. Whether the association of THRAP3 with the Mediator complex is regulated is not known. It was recently reported that THRAP3 can be phosphorylated on several serine residues [59]. Thus, it might be possible that the AP-1 activation pathway also leads to a phosphorylation-dependent association of THRAP3 with the Mediator complex, and a subsequent recruitment of the complex to AP-1-bound promoters. However, we cannot exclude the possibility that THRAP3 and the Mediator complex are both independently recruited to the promoter in an nTrip6-dependent manner. In such a scenario, THRAP3 would co-activate AP-1 independently from the action of the Mediator complex, possibly via the regulation of histone tail modification, since THRAP3 interacts with the lysine demethylase Jumonji [66].

### Mechanism of GR-mediated repression of AP-1 activity

Several mechanisms have been reported for the so-called crosstalk between GR and other transcription factors (reviewed in [67]–[69]). We have previously reported that nTRIP6 is essential in the negative crosstalk between GR and AP-1, in that it serves as an adaptor for the tethering of GR to the promoter-bound AP-1, which leads to transcriptional repression [19], [20]. We have now revealed a mechanism whereby the nTRIP6-dependent recruitment of GR leads to the inhibition of AP-1 activity. Given that nTRIP6 dimerization is essential for its co-activator function, one could have assumed that GR represses AP-1 by preventing the dimerization of nTRIP6. After ruling out this hypothesis, we showed that a competition between GR and THRAP3 for their interaction with nTRIP6 forms the basis of repression. While the second and third LIM domains of nTRIP6 are engaged in the interaction with the GR [19], [21], all three LIM domains participate in the interaction with THRAP3, indeed raising the possibility of a competition. Although there is a selectivity in the interaction between LIM domains and their binding partners, competitive binding has already been reported, for example in the case of LIM-only proteins (reviewed in [70]). Our results showing a decreased interaction between THRAP3 and nTRIP6 in the presence of GR confirmed that GR and THRAP3 compete for interaction with nTRIP6. Moreover, GR prevented the recruitment of THRAP3 to the activated MMP13 promoter, confirming that this competition occurs at the promoter of target genes. Given that THRAP3 is essential for the transcriptional activity of AP-1, the competition between THRAP3 and GR, resulting in an inhibition of THRAP3 promoter recruitment upon GR activation, most likely contributes to the GR-mediated repression of AP-1 activity. Finally, the observation that GR also inhibited the recruitment of Med1/TRAP220 further supports the idea that THRAP3 is the subunit responsible for the recruitment of the Mediator complex to AP-1-activated promoters. Thus, we propose that GR transrepresses AP-1 at least in part by displacing a THRAP3-containing Mediator complex from the promoter-bound nTRIP6-AP-1 complex.

**Figure 8:**
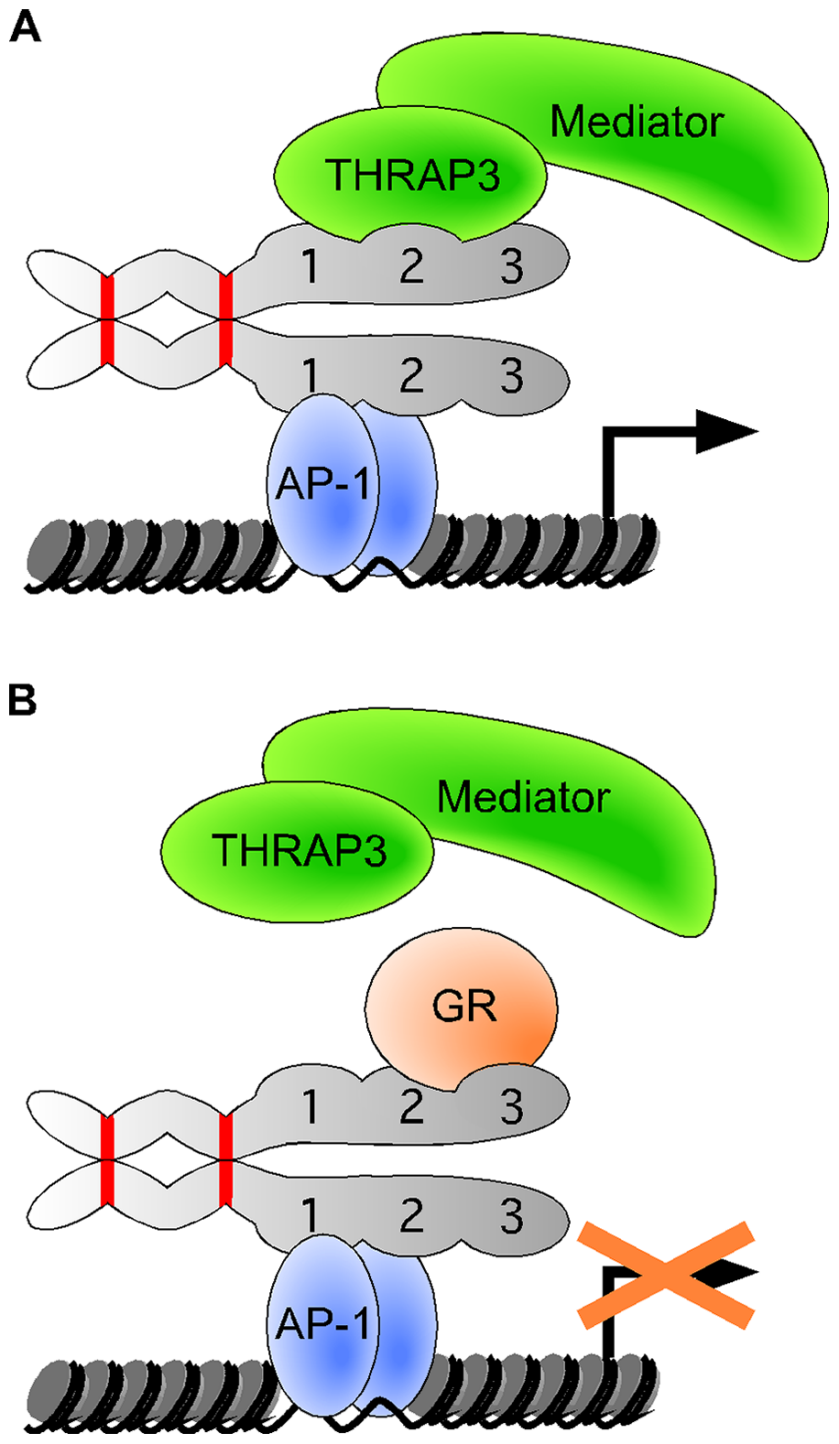
Model of nTRIP6-mediated regulation of AP1. nTRIP6 homodimerizes via 2 domains (depicted in red) within its N-terminal pre-LIM region. In activating conditions (**A**), nTRIP6 homodimers are recruited to the AP-1-bound promoter via an interaction of one set of LIM domains, and mediate the recruitment of THRAP3 and of the Mediator complex, via the other set of LIM domains. In repressing conditions (**B**), the glucocorticoid receptor (GR) is tethered to the promoter through an interaction with the LIM domains of nTRIP6, which prevents the recruitment of THRAP3 and of the Mediator complex.

In conclusion, we propose a model (Fig. 8) whereby nTRIP6 orchestrates the assembly of transcriptional co-regulators at AP-1-regulated promoters. Two domains in the N-terminal pre-LIM region of nTRIP6 mediate homodimerization, which enables one set of LIM domains to interact with promoter-bound AP-1, and the other set to recruit other co-activators such as THRAP3 and the Mediator complex. In the presence of glucocorticoids, the LIM domains-mediated tethering of GR prevents the recruitment of THRAP3 and the Mediator complex. Therefore, through the binding specificities of its multiple protein-protein interaction domains, nTRIP6 functions as a dual adaptor co-regulator, integrating both activating and repressing signals at the same transcription factor-bound promoter.

## Supporting Information

Figure S1 **Expression of the BiFC constructs.** HeLa cells were transfected with expression vectors for nTRIP6 fused to the N-terminal part of Venus (VN), for nTRIP6, nTRIP6 pre-LIM region lacking the 3 LIM domains (preLIM) or for only the 3 LIM domains (LIM) fused to the C-terminal part of Venus (VC). Cells were subjected to immunofluorescent labelling using an anti-HA antibody and counterstained with DRAQ5. Cells were imaged by confocal microscopy.
(TIF)

Figure S2 **Characterization of the AP-1-dependent reporter gene array cell line.** (**A**) Schematic representation of the AP-1-regulated gene unit amplified in the clone 12c. (**B**) Clone 12c and parental NIH-3T3 fibroblasts were subjected to DNA in situ hybridization using a fluorescently labelled cDNA probe complementary to the luciferase coding sequence (see Material and Methods S1). A single gene array is visible in the nucleus (delimited by dotted lines) of 100% of the 12c cells. (**C**) Clone 12c cells and parental NIH-3T3 fibroblasts were treated with solvent alone (con) or TPA as indicated. Luciferase activities are presented relatively to the untreated parental NIH-3T3 cells (mean ± S.D. of one representative experiment performed in triplicates). (**D**) nTRIP6 and RNA polymerase II are recruited to the gene array. 12c cells were transfected with GFP tagged RNA polymerase II (GFP-Pol2) or nTRIP6 fused to YFP. Cells were treated with solvent (con) or TPA for 3 h and imaged by confocal microscopy. (**E**) 12c cells were treated with solvent or TPA for 3 h, and endogenous c-Fos and Med1/TRAP220 were detected by immunofluorescence and confocal microscopy. Nuclei of representative cells are shown. The enrichment of RNA polymerase II, nTrip6, CBP and Med1/TRAP220 to the gene array upon TPA treatment (arrow) was observed in 70–80% of the transfected cells.
(TIF)

Figure S3 **Expression and localization of the BiFC fusions of nTRIP6 mutants lacking one dimerization domain.** HeLa cells were transfected with HA-tagged expression vectors for nTRIP6 or nTRIP6 lacking either the dimerization domain 1 (HA-nTRIP6ΔDD1) or the dimerization domain 2 (HA-nTRIP6ΔDD2), fused to either the N-terminal half (VN) or the C-terminal half (VC) of Venus. (**A**) Cell lysates were subjected to Western Blotting using an anti-HA antibody or an anti-GR antibody as a loading control. (**B**) Cells were subjected to immunofluorescent labelling using an anti-HA antibody, counterstained with DRAQ5, and imaged by confocal microscopy. (C) Representative images of the results in Fig. 2E. HeLa cells were cotransfected with the indicated combination of expression vectors for nTRIP6, nTRIP6ΔDD1 or nTRIP6ΔDD2, fused to either VN or VC, together with the mCherry-NES expression vector as a transfection control. Cells were imaged by confocal microscopy and representative cells are shown.
(TIF)

Figure S4 **Alignment of the N-terminal pre-LIM regions of proteins from the Zyxin family.** The residues corresponding to the dimerization domains (DD) 1 and 2 are boxed. The multiple sequence alignment was performed using the MultAlin software [71].
(TIF)

Figure S5 **The blocking peptide does not prevent the interaction between nTRIP6 and AP1.** HeLa cells were co-transfected with expression vectors for nTRIP6 fused to the N-terminal part of YFP (YN), and for the single chain AP-1 c-Jun∼c-Fos fused to the C-terminal part of YFP (YC), together with expression vectors for either mCherry fused to a nuclear localization signal (NLS; V), a peptide corresponding to the sequence of the dimerization domain 1 fused to an NLS and to mCherry (DD1), or a scrambled version of the DD1 peptide (DD1c). Cells were imaged by confocal microscopy. Representative images are shown in (**A**). In (**B**), YFP complementation was quantified by counting the number of transfected cells (mCherry positive) showing complementation (mean ± SD of three independent experiments).
(TIF)

Figure S6 **The DD1 peptide does not inhibit AP-1 when targeted to the cytosol.** (**A**) HEK293 cells were co-transfected with a luciferase reporter gene driven by the AP-1-dependent MMP1 promoter (MMP1-Luc) and Ubi-Renilla, together with either an expression vector for mOrange fused to a nuclear export signal (NES) as a control (V), or increasing amounts of an expression vector for the mOrange-NES fusion of the DD1 peptide (NES-DD1). Cells were treated with TPA as indicated. Normalized luciferase activities are plotted relative to the untreated vector control (mean ± SD of one representative experiment performed in triplicates). (**B**) HEK293 cells were transfected with the indicated constructs and imaged by confocal microscopy. Representative cells are shown.
(TIF)

Figure S7 **GR does not prevent nTRIP6 dimerization.** HeLa cells were co-transfected with nTRIP6 fused to the N-terminal part of Venus (VN) and nTRIP6 fused to the C-terminal part of Venus (VC), together with GR fused to mCherry. Cells were treated with dexamethasone (Dex) or solvent as a control (Con), and imaged 1 h later by confocal microscopy. Images of representative cells are shown.
(TIF)

Material and Methods S1 (DOCX)
