# Supplementary figures and images for: The LIM Domain Protein nTRIP6 Recruits the Mediator Complex to AP-1-Regulated Promoters
Source: PLoS One. 2014 May 12;9(5):e97549. doi: 10.1371/journal.pone.0097549 (PMC4018362; doi:10.1371/journal.pone.0097549)

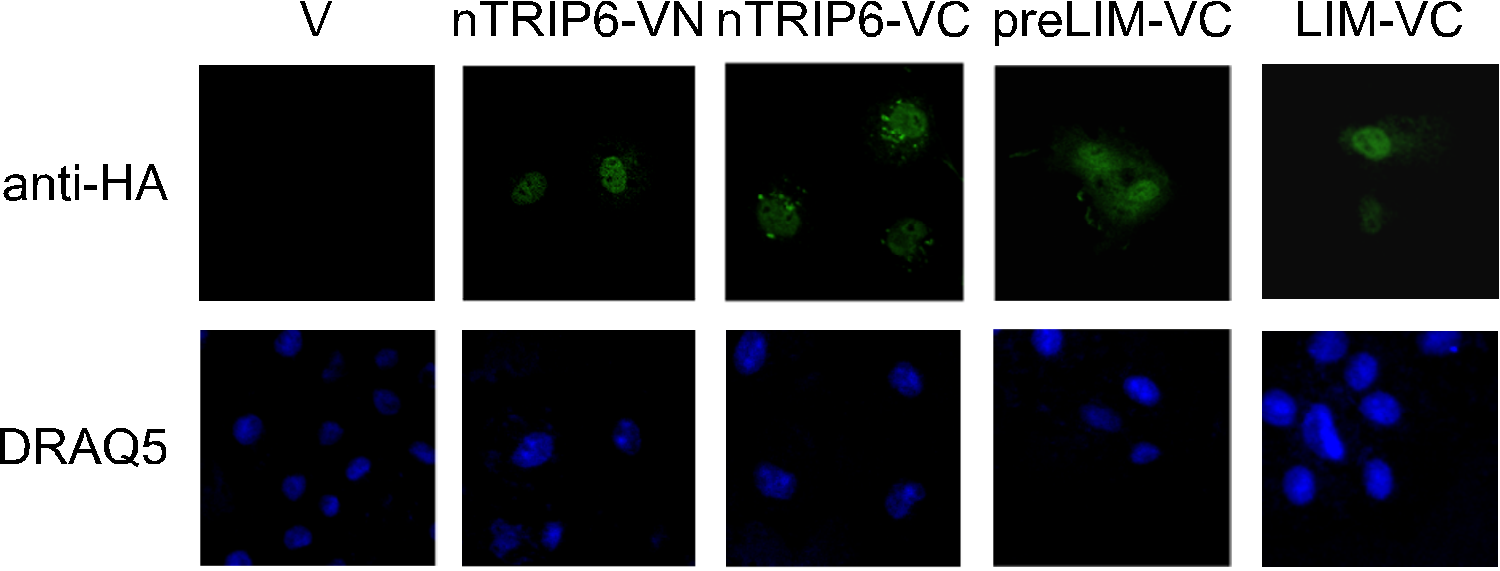

Supplement: Figure S1 — Expression of the BiFC constructs. HeLa cells were transfected with expression vectors for nTRIP6 fused to the N-terminal part of Venus (VN), for nTRIP6, nTRIP6 pre-LIM region lacking the 3 LIM domains (preLIM) or for only the 3 LIM domains (LIM) fused to the C-terminal part of Venus (VC). Cells were subjected to immunofluorescent labelling using an anti-HA antibody and counterstained with DRAQ5. Cells were imaged by confocal microscopy. (TIF) [file pone.0097549.s001.tif]

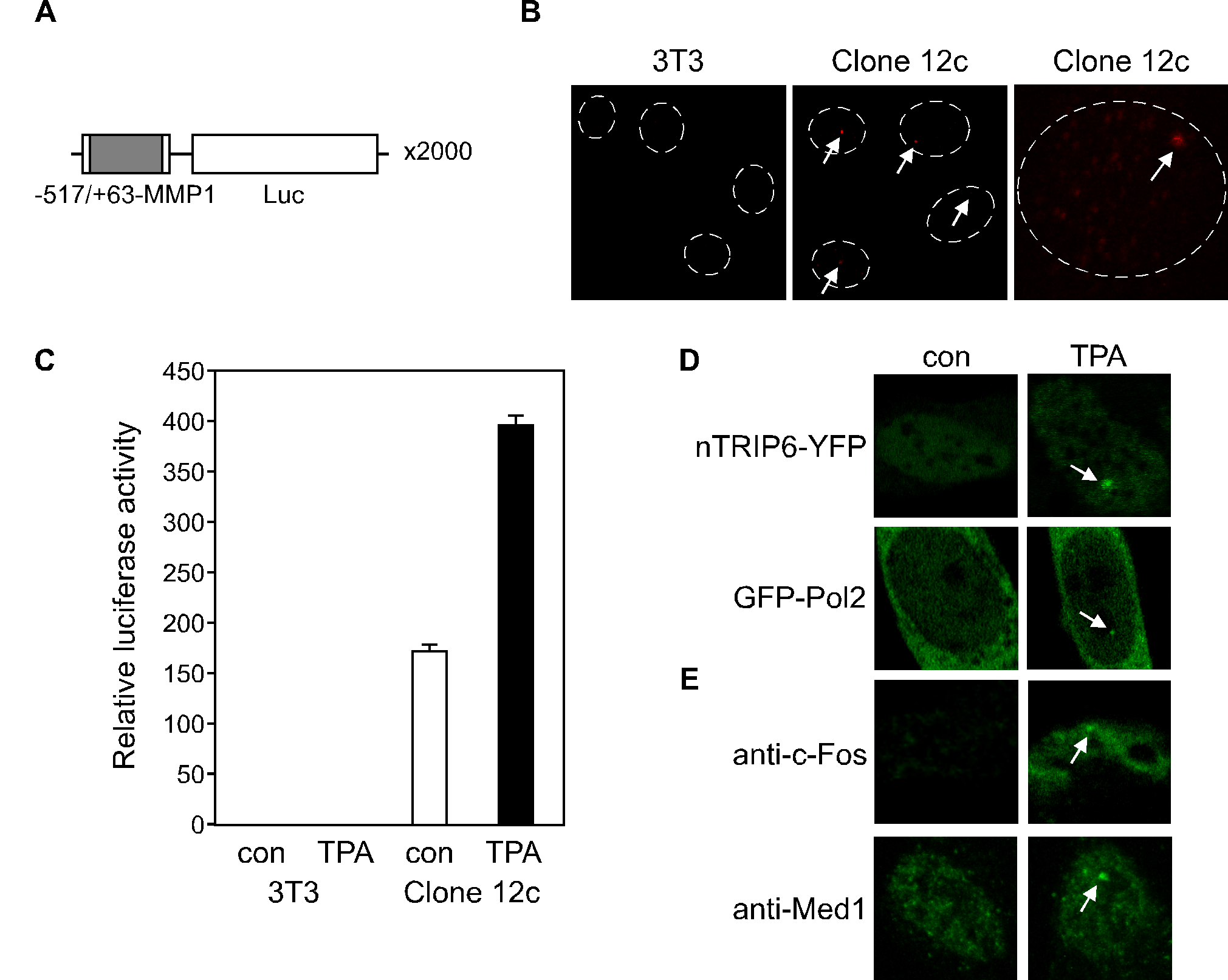

Supplement: Figure S2 — Characterization of the AP-1-dependent reporter gene array cell line. (A) Schematic representation of the AP-1-regulated gene unit amplified in the clone 12c. (B) Clone 12c and parental NIH-3T3 fibroblasts were subjected to DNA in situ hybridization using a fluorescently labelled cDNA probe complementary to the luciferase coding sequence (see Material and Methods S1). A single gene array is visible in the nucleus (delimited by dotted lines) of 100% of the 12c cells. (C) Clone 12c cells and parental NIH-3T3 fibroblasts were treated with solvent alone (con) or TPA as indicated. Luciferase activities are presented relatively to the untreated parental NIH-3T3 cells (mean ± S.D. of one representative experiment performed in triplicates). (D) nTRIP6 and RNA polymerase II are recruited to the gene array. 12c cells were transfected with GFP tagged RNA polymerase II (GFP-Pol2) or nTRIP6 fused to YFP. Cells were treated with solvent (con) or TPA for 3 h and imaged by confocal microscopy. (E) 12c cells were treated with solvent or TPA for 3 h, and endogenous c-Fos and Med1/TRAP220 were detected by immunofluorescence and confocal microscopy. Nuclei of representative cells are shown. The enrichment of RNA polymerase II, nTrip6, CBP and Med1/TRAP220 to the gene array upon TPA treatment (arrow) was observed in 70–80% of the transfected cells. (TIF) [file pone.0097549.s002.tif]

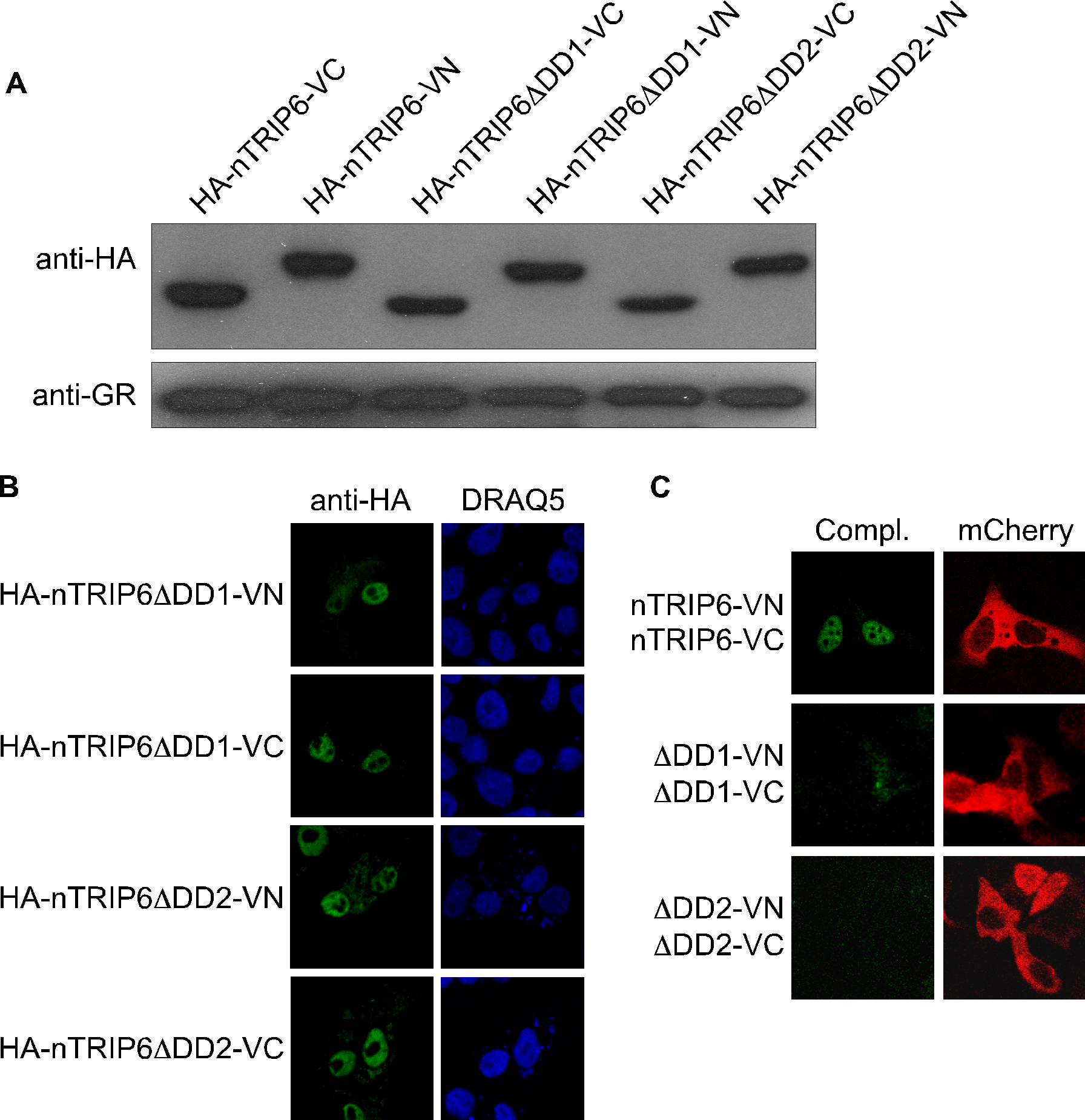

Supplement: Figure S3 — Expression and localization of the BiFC fusions of nTRIP6 mutants lacking one dimerization domain. HeLa cells were transfected with HA-tagged expression vectors for nTRIP6 or nTRIP6 lacking either the dimerization domain 1 (HA-nTRIP6ΔDD1) or the dimerization domain 2 (HA-nTRIP6ΔDD2), fused to either the N-terminal half (VN) or the C-terminal half (VC) of Venus. (A) Cell lysates were subjected to Western Blotting using an anti-HA antibody or an anti-GR antibody as a loading control. (B) Cells were subjected to immunofluorescent labelling using an anti-HA antibody, counterstained with DRAQ5, and imaged by confocal microscopy. (C) Representative images of the results in Fig. 2E. HeLa cells were cotransfected with the indicated combination of expression vectors for nTRIP6, nTRIP6ΔDD1 or nTRIP6ΔDD2, fused to either VN or VC, together with the mCherry-NES expression vector as a transfection control. Cells were imaged by confocal microscopy and representative cells are shown. (TIF) [file pone.0097549.s003.tif]

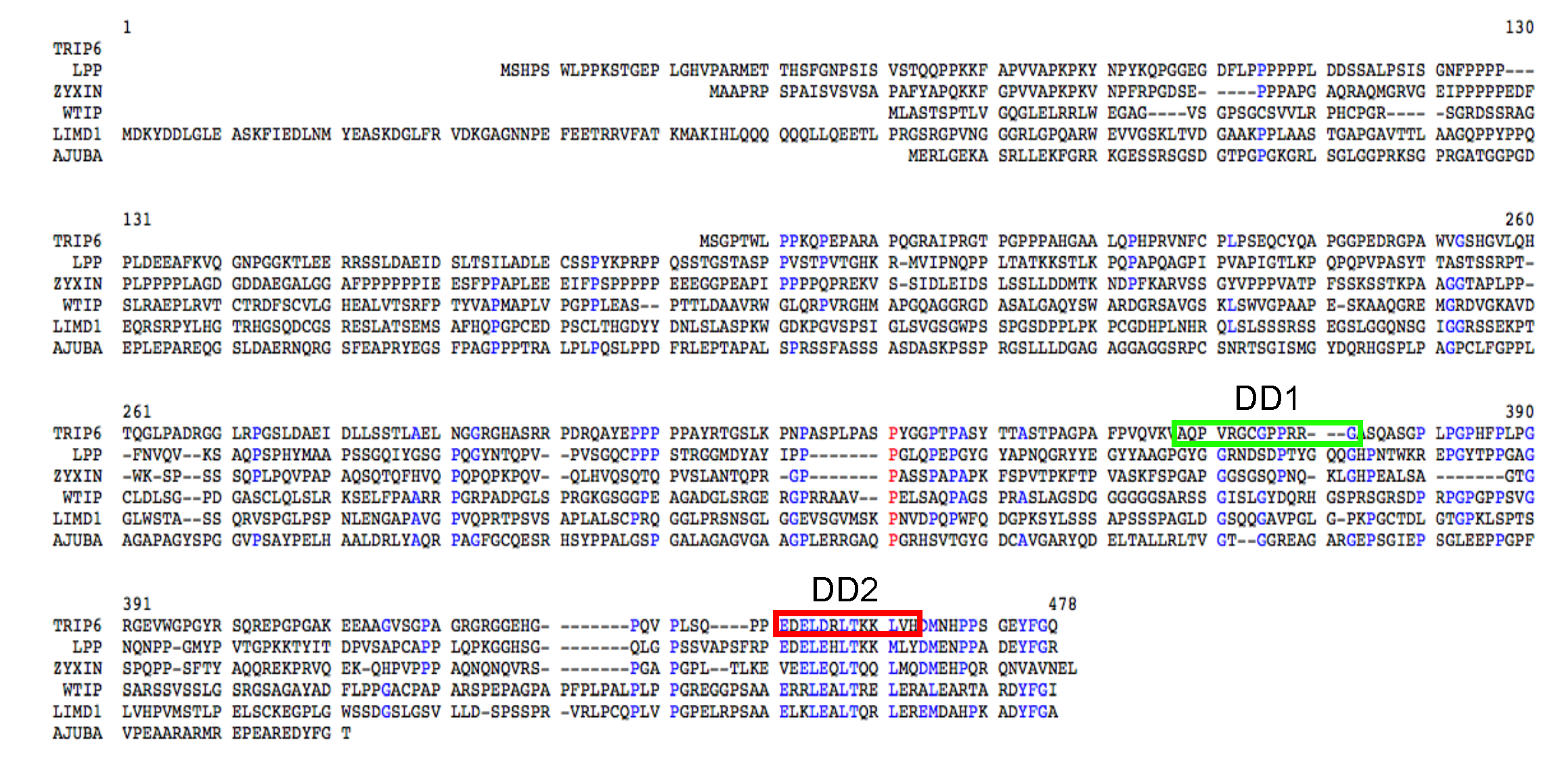

Supplement: Figure S4 — Alignment of the N-terminal pre-LIM regions of proteins from the Zyxin family. The residues corresponding to the dimerization domains (DD) 1 and 2 are boxed. The multiple sequence alignment was performed using the MultAlin software [71]. (TIF) [file pone.0097549.s004.tif]

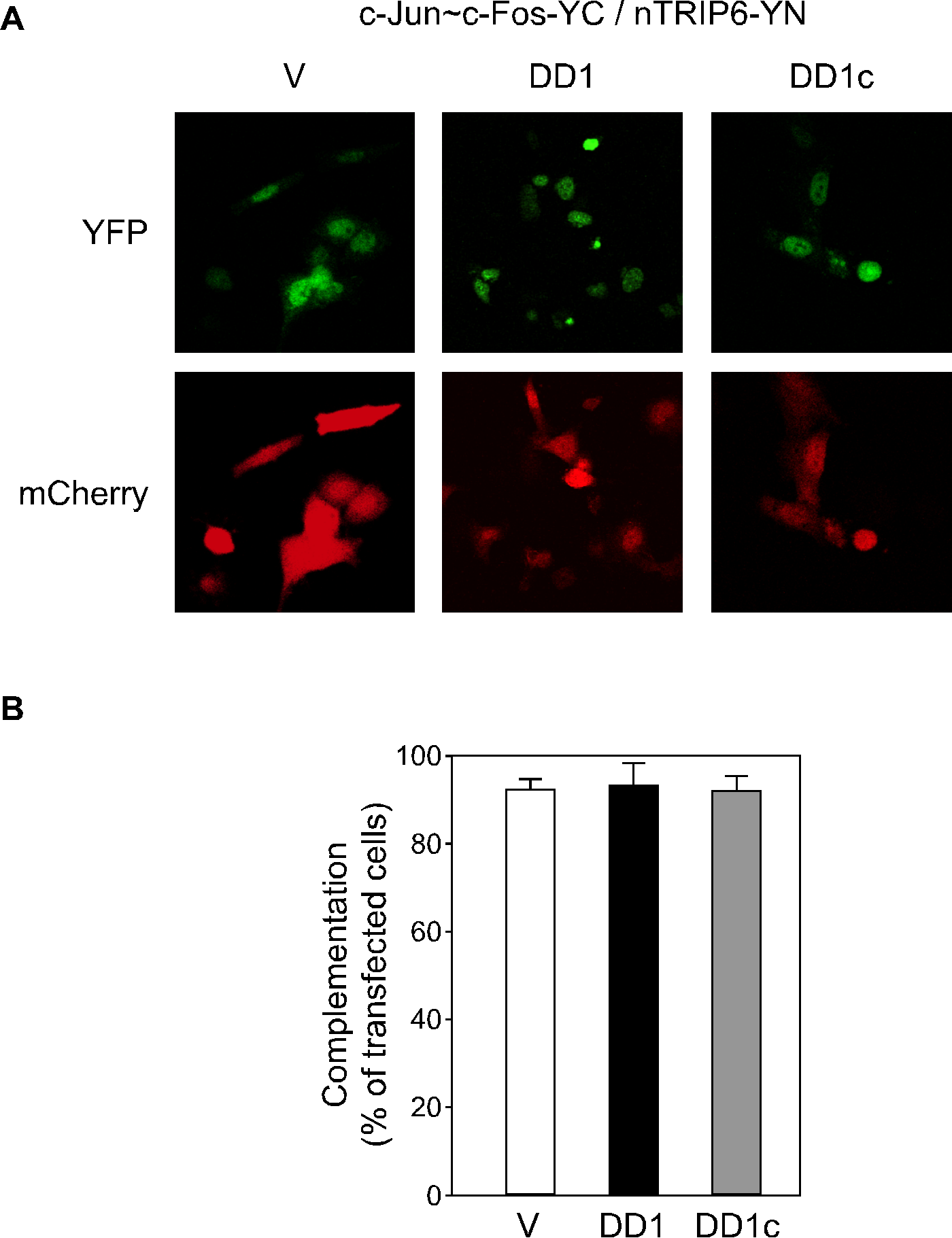

Supplement: Figure S5 — The blocking peptide does not prevent the interaction between nTRIP6 and AP1. HeLa cells were co-transfected with expression vectors for nTRIP6 fused to the N-terminal part of YFP (YN), and for the single chain AP-1 c-Jun∼c-Fos fused to the C-terminal part of YFP (YC), together with expression vectors for either mCherry fused to a nuclear localization signal (NLS; V), a peptide corresponding to the sequence of the dimerization domain 1 fused to an NLS and to mCherry (DD1), or a scrambled version of the DD1 peptide (DD1c). Cells were imaged by confocal microscopy. Representative images are shown in (A). In (B), YFP complementation was quantified by counting the number of transfected cells (mCherry positive) showing complementation (mean ± SD of three independent experiments). (TIF) [file pone.0097549.s005.tif]

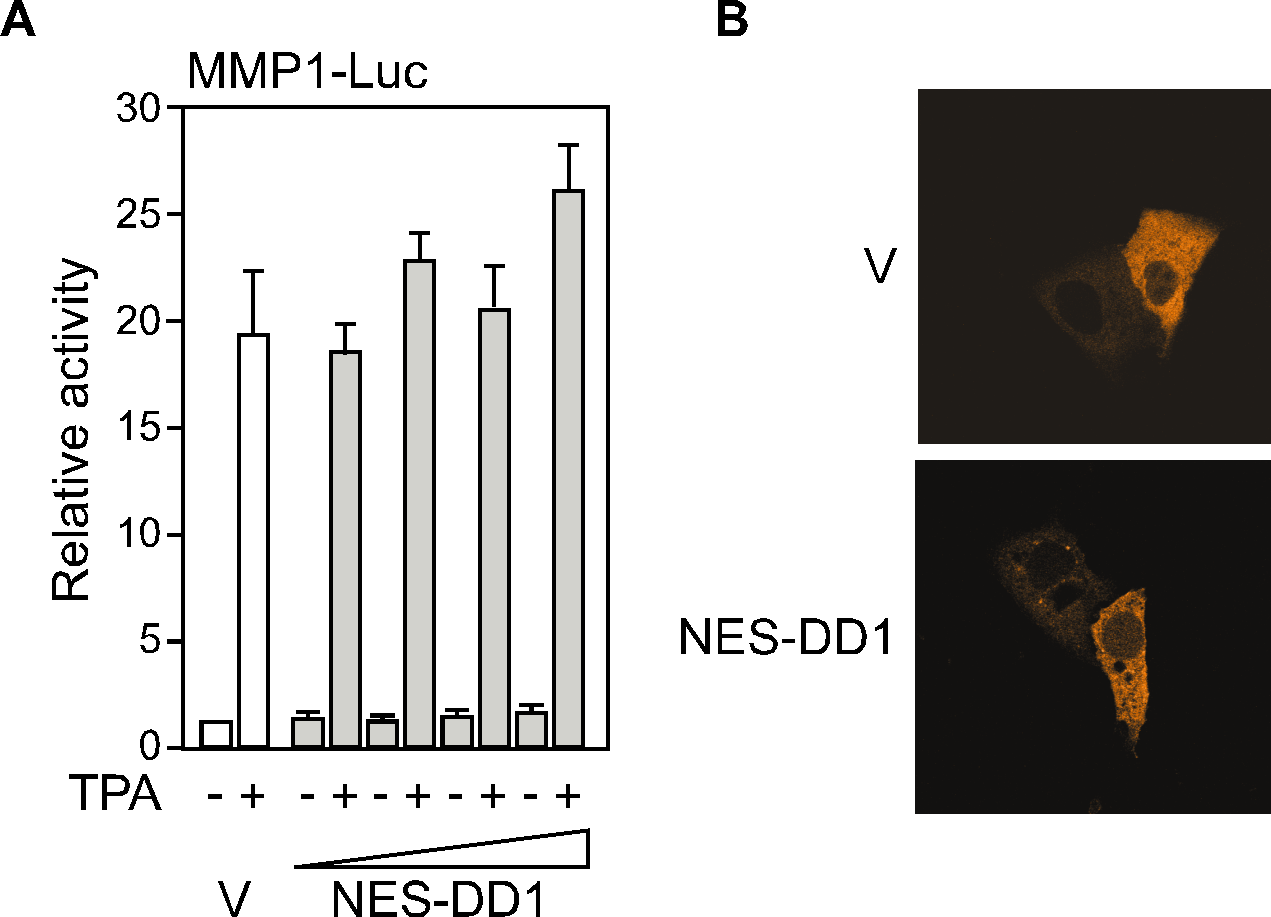

Supplement: Figure S6 — The DD1 peptide does not inhibit AP-1 when targeted to the cytosol. (A) HEK293 cells were co-transfected with a luciferase reporter gene driven by the AP-1-dependent MMP1 promoter (MMP1-Luc) and Ubi-Renilla, together with either an expression vector for mOrange fused to a nuclear export signal (NES) as a control (V), or increasing amounts of an expression vector for the mOrange-NES fusion of the DD1 peptide (NES-DD1). Cells were treated with TPA as indicated. Normalized luciferase activities are plotted relative to the untreated vector control (mean ± SD of one representative experiment performed in triplicates). (B) HEK293 cells were transfected with the indicated constructs and imaged by confocal microscopy. Representative cells are shown. (TIF) [file pone.0097549.s006.tif]

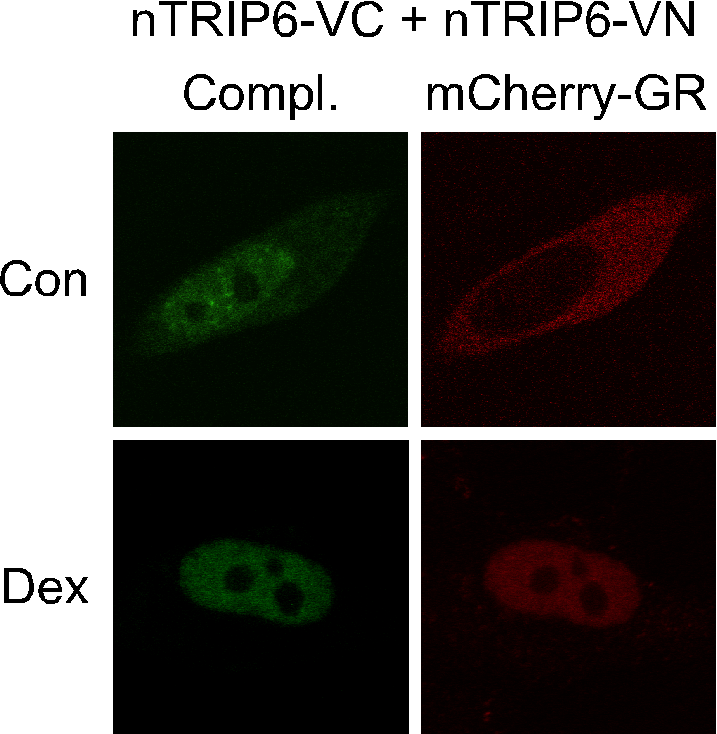

Supplement: Figure S7 — GR does not prevent nTRIP6 dimerization. HeLa cells were co-transfected with nTRIP6 fused to the N-terminal part of Venus (VN) and nTRIP6 fused to the C-terminal part of Venus (VC), together with GR fused to mCherry. Cells were treated with dexamethasone (Dex) or solvent as a control (Con), and imaged 1 h later by confocal microscopy. Images of representative cells are shown. (TIF) [file pone.0097549.s007.tif]
