# Supplementary material for: The LIM Domain Protein nTRIP6 Recruits the Mediator Complex to AP-1-Regulated Promoters
Source: PLoS One. 2014 May 12;9(5):e97549. doi: 10.1371/journal.pone.0097549 (PMC4018362; doi:10.1371/journal.pone.0097549)
Supplement: Material and Methods S1 — (DOCX) [file pone.0097549.s008.docx]

Material and Methods S1 (pertaining to supplementary figures)

Plasmids constructs

pEGFP-Pol II, encoding an EGFP fusion of the largest subunit of RNA polymerase II [70], was from Kimihiko Sugaya (National Institute of Radiological Sciences Chiba, Japan). nTRIP6 fused to YFP, nTRIP6 fused to the N-terminal part of YFP (YN), and the single chain AP-1 dimer c-Jun~c-Fos fused to the C-terminal part of YFP (YC) have been described [20]. The mOrange-tagged, cytosol targeted blocking peptide DD1 was obtained by cloning mOrange between the NheI and KpnI sites, an oligonucleotide encoding the nuclear export signal (NES) of MEK1 between the KpnI and BamHI sites, and an oligonucleotide encoding the peptide between the BamHI and XbaI sites of pcDNA3.1. In pcDNA3.1mCherry-GR, mCherry and GR sequences were PCR amplified and cloned in frame into pcDNA3.1.

DNA in situ hybridization

The presence of the amplified Coll-Luc array in clone 12c was confirmed by fluorescence in situ hybridization (FISH), as described [20,21]. Briefly, cells grown on coverslips were fixed for 30 min at room temperature in 3.5% paraformaldehyde, permeabilized in 0.5% Triton X-100 in PBS for 10 min, and treated with RNAse (50 µg/ml) for 1 h. DNA was denatured in 50% formamide/2X standard saline citrate (SSC) at 95°C for 5 min, then on ice for 5 min. Cells were then dehydrated on ice in 70, 90 then 100% ethanol for 5 min each. A fluorescent probe targeting the luciferase coding sequence was generated by PCR in the presence of chromatide Alexa 546 dUTP (Invitrogen). The purified probe was denatured in 25% formamide at 95°C for 5 min, and diluted at 5-10 µg/ml in hybridization solution (50% formamide, 2X SSC, 10% dextran sulfate, 1 mg/ml *Escherichia coli* tRNA). Hybridization was performed at 37°C for 16 h, followed by washes in 2X SSC/0.05% Triton X-100 for 10 min, in 2X SSC for 15 min, and then in 4X SSC for 5 min. Imaging was performed by laser scanning microscopy.

Multiple sequence alignment

Multiple sequence alignments of the N-terminal pre-LIM regions of proteins from the Zyxin family were performed using the MultAlin software [71] available at http://multalin.toulouse.inra.fr.
